# Pulsatile desynchronizing delayed feedback for closed-loop deep brain stimulation

**DOI:** 10.1371/journal.pone.0173363

**Published:** 2017-03-08

**Authors:** Oleksandr V. Popovych, Borys Lysyansky, Michael Rosenblum, Arkady Pikovsky, Peter A. Tass

**Affiliations:** 1 Institute of Neuroscience and Medicine - Neuromodulation, Jülich Research Center, Jülich, Germany; 2 Institute of Physics and Astronomy, University of Potsdam, Potsdam-Golm, Germany; 3 Department of Neurosurgery, Stanford University, Stanford, California, United States of America; 4 Department of Neuromodulation, University of Cologne, Cologne, Germany; Universidad Rey Juan Carlos, SPAIN

## Abstract

High-frequency (HF) deep brain stimulation (DBS) is the gold standard for the treatment of medically refractory movement disorders like Parkinson’s disease, essential tremor, and dystonia, with a significant potential for application to other neurological diseases. The standard setup of HF DBS utilizes an open-loop stimulation protocol, where a permanent HF electrical pulse train is administered to the brain target areas irrespectively of the ongoing neuronal dynamics. Recent experimental and clinical studies demonstrate that a closed-loop, adaptive DBS might be superior to the open-loop setup. We here combine the notion of the adaptive high-frequency stimulation approach, that aims at delivering stimulation adapted to the extent of appropriately detected biomarkers, with specifically desynchronizing stimulation protocols. To this end, we extend the delayed feedback stimulation methods, which are intrinsically closed-loop techniques and specifically designed to desynchronize abnormal neuronal synchronization, to pulsatile electrical brain stimulation. We show that permanent pulsatile high-frequency stimulation subjected to an amplitude modulation by linear or nonlinear delayed feedback methods can effectively and robustly desynchronize a STN-GPe network of model neurons and suggest this approach for desynchronizing closed-loop DBS.

## Introduction

Synchronization is a fundamental natural phenomenon in interacting networks [1–4]. Synchronization plays a crucial role in the human brain in, e.g., processing of sensory information [5], motor control [6], and cognitive function [7]. However, excessive pathological neuronal synchrony may severely impair brain function and is a hallmark of several neurological disorders, such as Parkinson’s disease (PD) [8, 9], essential tremor [10], epilepsy [11], and tinnitus [12–14]. The standard therapy for the treatment of medically refractory PD is high frequency (HF) deep brain stimulation (DBS), where electrical HF pulse trains are administered at frequencies >100 Hz via depth electrodes chronically implanted in target areas such as the thalamic ventralis intermedius (VIM) nucleus, the subthalamic nucleus (STN), or the globus pallidus (GP) [15–18]. HF DBS has been developed empirically, and the clinical and electrophysiological mechanisms of the symptom suppression by HF DBS are still a matter of intensive research [17, 19, 20]. A large number of studies are devoted to an improvement of the therapeutic effects of HF DBS by appropriate calibration of the stimulation parameters such as stimulation frequency and intensity, the width and shape of the stimulation pulses, spatial spread and localization of the stimulation current in the neuronal tissue, as well as selection of appropriate stimulation targets, etc. [15–17, 21–25]. A key aspect for further improvement of DBS is the reduction of side effects. HF DBS may not only cause side effects by the spread of electrical current outside of the target region, but also by chronic stimulation of the target itself as well as due to functional disconnection of the stimulated structure [26–29]. Hence, it is crucial to reduce the integral current required.

In contrast to the standard open-loop HF DBS, the major goal of closed-loop, demand controlled DBS is to stimulate only when necessary and/or to adapt the strength of stimulation to the amount of abnormal neuronal synchrony. Demand-controlled DBS was initially introduced in computational studies with different types of specifically designed desynchronizing stimuli and different types of closed-loop control modes, e.g. demand-controlled timing of stimulus delivery or demand-controlled adaptation of stimulus strength during period stimulus delivery [30–32]. So far demand-controlled DBS was experimentally tested by means of conventional high-frequency stimulation and denoted as adaptive DBS (aDBS) [33–36]. In monkeys rendered parkinsonian with the neurotoxin 1-methyl-4-phenyl-1,2,3,6-tetrahydropyridine (MPTP), closed-loop DBS was tested under acute conditions [37], where a short stimulation pulse train (7 pulses at 130 Hz) was delivered through a pair of electrodes located in the globus pallidus internal (GPi) with an optimal time delay of 80 ms following the occurrence of an action potential recorded either from the GPi or the primary motor cortex (M1). This type of stimulation caused a strong decrease of the firing rate of pallidal neurons together with a pronounced decrease of the oscillatory neuronal activity at the tremor frequency (4−7 Hz) and at the double tremor frequency (9−15 Hz) along with an amelioration of the MPTP-induced akinesia [37]. In contrast, standard continuous 130 Hz DBS caused a less pronounced decrease of the pallidal firing rate, the oscillatory neuronal activity and the amelioration of the akinesia [37].

Another study reported on a successful proof of principle of a closed-loop aDBS in PD patients, where the onsets and offsets of HF DBS were triggered by threshold crossings by local field potential (LFP) assessing beta-band STN activity [34]. The onset of the stimulation was delayed by 30 to 40 ms with respect to the threshold crossing by LFP. The average improvement in clinical motor scores in the aDBS condition was significantly better by about 30% despite delivering less than 50% of the stimulation current as compared to the conventional continuous HF DBS (cDBS) condition [34]. Clinical and electrophysiological (suppressing of beta-band LFP oscillations) effects of aDBS were also stronger compared to the intermittent random DBS, where random DBS bursts were not triggered by the LFP threshold crossings. This indicates that the intermittency itself is not a key determinate of the efficacy observed with aDBS, and the real-time feedback from the ongoing LFP oscillations is necessary [34].

The on-demand DBS was applied for suppression of essential tremor [38], where the electromyographic (EMG) signal was used to predict the onset of the tremor and initiation of the DBS epoch lasting for a few tens of seconds. Tremor was suppressed by such an intermittent stimulation, which indicated the feasibility of the EMG-based predictive on-off control of DBS in essential tremor patients. The on-demand closed-loop DBS was also tested in patients with intention tremor, where the HF DBS was switched on and off when the power of the recorded EMG activity exceeded or decreased below the EMG trigger threshold, respectively [39]. The on-demand control system triggered the switching on/off of DBS accurately and controlled intention tremor completely.

Closed-loop aDBS also proved to be superior to conventional open-loop DBS (cDBS) in a freely moving patient with PD stimulated with aDBS up to 6 days after DBS electrode implantation [35]. During aDBS the patient experienced a more stable condition with better control of symptoms and dyskinesia as well as improvement of bradykinesia than during cDBS. Instead of the on-off strategy of the papers [34, 37–39], the stimulation voltage was linearly adapted each second based on the LFP beta band power [35]. When the beta-band LFP activity was reduced, the voltage got diminished, in this way avoiding unnecessary hyperstimulation, and the aDBS did not elicit side effects and was well tolerated.

Another direction of research is the model-based development of novel stimulation techniques aiming at specifically counteracting abnormal neuronal synchrony by desynchronization [3]. Initially these techniques were designed to achieve a demand-controlled desynchronizing stimulation [30–32]. However, by taking into account spike timing-dependent plasticity (STDP) [40, 41] in the model neural networks, computationally it was shown that coordinated reset (CR) stimulation [32], a spatio-temporally patterned, desynchronizing stimulation technique developed in the framework of the model-based approach, reduces the rate of coincidences and, hence, down-regulates abnormal synaptic weights [42, 43]. In this way cumulative, long-lasting, sustained desynchronizing effects can be obtained [42, 43]. Based on numerous modeling studies, that formed a solid foundation for the application of CR stimulation with different stimulation modalities [32, 42, 44–46], these computational predictions were verified both pre-clinically and clinically.

Long-lasting CR-induced desynchronization was confirmed *in vitro* in rat hippocampal slices rendered epileptic by magnesium withdrawal [47]. CR stimulation caused beneficial therapeutic long-lasting after-effects in parkinsonian MPTP-treated monkeys [48, 49] and in PD patients [50], whereas the standard HF DBS only has acute effects, i.e., neither clinical [17, 51] nor electrophysiological [52, 53] effects reliably persisted after cessation of stimulation. Based on the same principles of desynchronization-induced unlearning of abnormal synaptic connectivity and neuronal synchrony, i.e. anti-kindling [42], non-invasive, acoustic CR stimulation was successively applied in a clinical proof of concept study in tinnitus patients [54]. In accordance with the results of modeling studies [44, 45], acoustic CR stimulation can significantly counteract both tinnitus symptoms and the underlying pathological neuronal synchronization by normalizing the effective connectivity and restoring the functional patterns of activity [54].

In line with the above promising model-based development of desynchronizing CR stimulation, other methods have been developed for the control of abnormal neuronal synchronization. They are based on feedback techniques, where the mean field of synchronized population is measured, preprocessed and fed back as stimulation signal [55–64], or on phase response properties of neurons, where the stimulation signal can be derived from the phase response curve (PRC) [65–67]. The feedback methods can be applied under a variety of conditions and possess an intrinsic demand-controlled character, where the stimulation signal is significantly reduced or even vanishes as soon as desynchronization is achieved. These methods have in common that the stimulation current is a smooth and slowly oscillating signal (although the PRC approach allows for pulsatile stimuli as well), whereas the standard HF DBS and CR stimulation utilize trains of short charge-balanced pulses [22, 68]. The experimental and clinical realization of these feedback methods is a challenging task, first of all, from the technical side, since stimulation signals have to fulfill all safety aspects like charge density limits [16, 69, 70]. These charge density limits actually crucially restrict the applicability of the slow feedback signals. As opposed to the timescales of HF DBS and CR pulses, the feedback signals are slow, so that, if the feedback signal is used directly for stimulation, during the comparably long feedback stimulation periods an irreversible charge deposit can exceed safety limits.

In this paper we resolve this problem and show that the desynchronizing impact and the demand-controlled character of feedback techniques from one side can be combined with the advantages of the charge-balanced property of the HF DBS signal from another side. For this, the amplitude of the HF train of charge-balanced pulses is modulated by the slow feedback signal, which represents *a pulsatile feedback stimulation* appropriate for electrical DBS. We consider two feedback stimulation methods, linear delayed feedback (LDF) [55, 56] and nonlinear delayed feedback (NDF) [57, 61], and illustrate their efficacy in desynchronizing the model network of STN-GPe neurons suggested in the papers [71, 72]. We administer the delayed feedback stimulations to strongly or weakly and intermittently synchronized neuronal populations and demonstrate that they have a robust desynchronizing impact with respect to parameter variation.

In this way we question the main conclusion of the paper by Dovzhenok *et al*. [73] considering the same model and claiming that the delayed feedback DBS is unlikely to be clinically successful because the delayed feedback can desynchronize strongly synchronized neurons, but the same feedback can boost synchronization for intermittently synchronized neurons. This claim is disorienting and requires clarification, because it depreciates the great amount of modeling [55–57, 61, 63, 74] and experimental [75, 76] studies of LDF and NDF as well as recent promising experimental and clinical attempts to advance standard HF DBS to a closed-loop stimulation setup, which attracts the attention of many researchers [34, 35, 37–39, 77–80]. We numerically demonstrate that the pulsatile LDF and NDF are equally effective in counteracting synchronization in strongly as well as weakly and intermittently synchronized neuronal populations. The feedback techniques (LDF, NDF) with smooth stimulation signal are also effectively causing desynchronization in our model. To this end, however, safety requirements for electrical stimulation of neuronal tissue have to be satisfied [16, 69, 70]. Hence, based on our computational study we suggest to use pulsatile LDF and NDF to achieve desynchronizing effects, thereby preserving mandatory safety requirements. In this paper we disprove the argumentation by Dovzhenok *et al*. [73]. However, more importantly, we suggest a safe solution that paves the way for properly modified DBS feedback techniques to be applied in a first in man study. Our results contribute to the growing field of adaptive closed-loop DBS and show that the suggested approach of pulsatile delayed feedback stimulation could be a possible enhancement of the standard HF DBS, where an abnormal neuronal synchronization can reliably be suppressed by a minimal amount of stimulation.

## Methods

### Model

We consider a network of two neuronal populations, which models the dynamics of STN and GPe neurons. Each cell is modeled by the following system [71]:

$$\begin{array}{ccc} C_{m}v^{\prime } & = & -I_{\text{L}}-I_{\text{K}}-I_{\text{Na}}-I_{\text{T}}-I_{\text{Ca}}-I_{\text{AHP}}-I_{\text{syn}}+I_{\text{app}}+I_{\text{stim}}, \end{array}$$ (1)

$$\begin{array}{ccc} {\left[\text{Ca}\right]}^{\prime } & = & \varepsilon \left(-I_{\text{Ca}}-I_{\text{T}}-k_{\text{Ca}}\left[\text{Ca}\right]\right), \end{array}$$ (2)

$$\begin{array}{ccc} X^{\prime } & = & \phi_{X}\left(X_{\infty }\left(v\right)-X\right)/\tau_{X}\left(v\right), \end{array}$$ (3)

where *v* is a membrane potential of the neuron, *I*_L_, *I*_K_, *I*_Na_, *I*_T_, *I*_Ca_, *I*_AHP_, *I*_syn_, and *I*_app_ are the corresponding leak, potassium, sodium, low threshold calcium, high threshold calcium, afterhyperpolarisation potassium, synaptic, and external current, respectively. The stimulation current *I*_stim_ will be defined below. [Ca] is the intracellular concentration of Ca_2+_ ions, and *X* = *n*, *h*, *r* are the gating variables.

The following currents from Eq (1) attain the same form for both types of neurons:

$$\begin{array}{c} \begin{array}{cc} I_{\text{L}}=g_{\text{L}}\left(v-v_{\text{L}}\right), & I_{\text{K}}=g_{\text{K}}n^{4}\left(v-v_{\text{K}}\right),\\ I_{\text{Na}}=g_{\text{Na}}m_{\infty }^{3}\left(v\right)h\left(v-v_{\text{Na}}\right), & I_{\text{Ca}}=g_{\text{Ca}}s_{\infty }^{2}\left(v\right)\left(v-v_{\text{Ca}}\right),\\ I_{\text{AHP}}=g_{\text{AHP}}\left(v-v_{\text{K}}\right)(\left[\text{Ca}\right]/(\left[\text{Ca}\right]+{\text{K}}_{1})), \end{array} \end{array}$$

whereas current *I*_T_ is given by different expressions for the excitatory STN cells and for the inhibitory GPe cells:

$$\begin{array}{c} \text{STN}:\;\;I_{\text{T}}=g_{\text{T}}a_{\infty }^{3}\left(v\right)b_{\infty }^{2}\left(r\right)\left(v-v_{\text{Ca}}\right),\;\;\text{GPe}:\;\;I_{\text{T}}=g_{\text{T}}a_{\infty }^{3}\left(v\right)r\left(v-v_{\text{Ca}}\right), \end{array}$$

where *b*_∞_(*r*) = 1/(1 + exp[(*r* − *θ*_*b*_)/*σ*_*b*_]) − 1/(1 + exp[−*θ*_*b*_/*σ*_*b*_]). The functions *X*_∞_(*v*) and *τ*_*X*_(*v*) used in Eq (3) and in the above definition of the currents read

$$\begin{array}{c} \begin{array}{cccc} X_{\infty }\left(v\right) & = & 1/\left(1+\exp[-\left(v-\theta_{X}\right)/\sigma_{X}]\right), & X=n,\;h,\;r,\;m,\;s,\;a,\\ \tau_{X}\left(v\right) & = & \tau_{X}^{0}+\tau_{X}^{1}/\left(1+\exp[-\left(v-\theta_{X}^{\tau }\right)/\sigma_{X}^{\tau }]\right),\; & X=n,\;h,\;r. \end{array} \end{array}$$

For GPe neurons *τ*_*r*_(*v*) = *τ*_*r*_ is a constant parameter.

**Fig 1:**
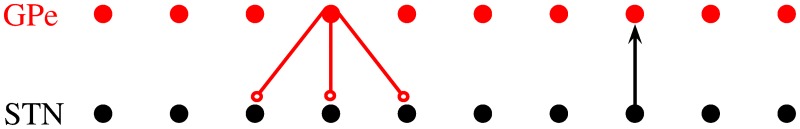
Coupling pattern of the STN-GPe neuronal network. Black circles depict STN cells, red circles depict GPe neurons. Each STN neuron excites a single GPe cell, whereas each GPe cell inhibits three STN neurons.

In our study we consider coupled populations of *N* = 200 STN and 200 GPe neurons. The STN and GPe neuronal ensembles and coupling among them are schematically illustrated in Fig 1. Each STN neuron excites a single GPe neuron, whereas each GPe neuron inhibits three neighboring STN neurons. We also consider periodic boundary conditions. Microscopic models of this type were introduced and investigated in a number of papers [71–73, 81], where STN neurons receive an inhibitory input from GPe neurons and, in turn, give an excitatory output to the GPe network.

The coupling among the neurons is realized via synaptic currents *I*_syn_ defined in the following way:

$$\begin{array}{c} \text{STN}:\;\;I_{\text{syn}}=g_{\text{G}\to S}\left(v-v_{\text{G}\to S}\right)\sum s_{\text{j}},\;\;\;\text{GPe}:\;\;I_{\text{syn}}=g_{\text{S}\to G}\left(v-v_{\text{S}\to \text{G}}\right)\sum s_{\text{j}}, \end{array}$$

for STN and GPe cells, respectively. *j* is the index of neurons and summations are taken over all presynaptic neurons. The synaptic weights *g*_S→G_ = 0.4 nS/*μ*m^2^ and *g*_G→S_ = 1.7 nS/*μ*m^2^ reflect the strength of the coupling from STN neurons to GPe neurons, and in the opposite direction, respectively. We also consider a weaker coupling *g*_G→S_ = 1.28 nS/*μ*m^2^. The reversal potentials *v*_S→G_ = 0 mV and *v*_G→S_ = −100 mV reflect the excitatory coupling from STN to GPe neurons and inhibitory coupling from GPe to STN, respectively. The equation for the synaptic variables *s*_*j*_ reads:

$$\begin{array}{c} s_{j}^{\prime }=\alpha H_{\infty }\left(v_{j}-\theta_{g}\right)\left(1-s_{j}\right)-\beta s_{j},\;H_{\infty }\left(x\right)=1/\left(1+\exp\left[-\left(x-\theta_{g}^{H}\right)/\sigma_{g}^{H}\right]\right). \end{array}$$ (4)

We suppose that the neurons in the STN and GPe ensembles are nonidentical. For this, the applied currents *I_app_* = *I*_app_, *j* for STN cells are Gaussian distributed with the mean 10 pA/*μ*m^2^ and the standard deviation 0.015 pA/*μ*m^2^. The parameter *ε* = *ε*_*j*_ for GPe neurons are also Gaussian distributed with the mean 0.0055 ms^−1^ and the standard deviation 2 ⋅ 10^−5^ ms^−1^. The values of the other parameters for the STN and GPe neurons are given in S1 Appendix.

**Fig 2:**
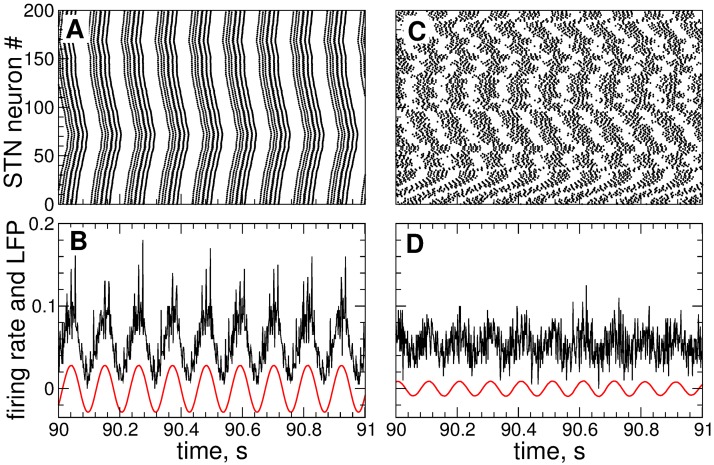
Intrinsic dynamics of the STN neurons Eqs (1)–(3) without stimulation. The raster plots (A) and (C) and firing rates (relative number of neurons firing a spike at a given time) in plots (B) and (D) illustrate the STN neuronal activity in a strongly synchronized regime (A), (B) with the coupling *g*_G→S_ = 1.7 nS/*μ*m^2^ from GPe to STN and in a weakly synchronized regime (C), (D) with *g*_G→S_ = 1.28 nS/*μ*m^2^. The red solid curves in plots (B) and (D) depict the filtered LFP. Stimulation current *I*_stim_ = 0.

In Fig 2 we illustrate the dynamics of STN neurons for the considered sets of parameters. For a strong coupling *g*_G→S_ = 1.7 nS/*μ*m^2^ the STN neurons synchronize and fire bursts nearly simultaneously [Fig 2A]. Such a synchronized dynamics of individual neurons results in a rhythmic activity of the STN as reflected, for example, by the collective firing rate [Fig 2B], which is the relative number of neurons firing a spike at a given time. The local field potential (LFP, see below for definition) of synchronized STN neurons also demonstrates well-pronounced oscillations of large amplitude [Fig 2B, red solid curve], which can serve as an indicator of a synchronized neuronal dynamics. For a weak coupling *g*_G→S_ = 1.28 nS/*μ*m^2^ the neurons are much less synchronized [Fig 2C], and STN does not produce a pronounced rhythmic output as illustrated by the firing rate in Fig 2D. The same applies to the dynamics of the LFP which exhibits low-amplitude oscillations [Fig 2D, red solid curve] indicating a desynchronized dynamics of the individual neurons.

In this study we focus on the control of the collective synchronized dynamics of the STN-GPe network. The extent of synchronization can be estimated either by the LFP amplitude [Fig 2B and 2D, red solid curves] or by the order parameter $R\left(t\right)=|N^{-1}\sum_{j=1}^{N}\exp\left(i\psi_{j}\left(t\right)\right)|$ [2, 3, 82], where *ψ*_*j*_(*t*) are the phases of individual neurons calculated from the neuronal bursting dynamics. The phase *ψ*_*j*_(*t*) of the *j*th neuron attains the values *ψ*_*j*_(*t*_*n*_) = 2*πn*, *n* = 0, 1,… at the time moments *t*_*n*_ of the burst onsets, i.e., the first spikes in the bursts, and linearly increases between the neighboring bursts *ψ*_*j*_(*t*) = 2*π*(*t* − *t*_*n*_)/(*t*_*n*+1_ − *t*_*n*_) + 2*πn* for *t* ∈ (*t*_*n*_, *t*_*n*+1_), *n* = 0, 1,… [4]. The order parameter *R*(*t*) ranges from 0 to 1, where 1 corresponds to a perfect in-phase synchronization and 0 indicates a desynchronized state. For example, the time-averaged order parameter 〈*R*(*t*)〉 ≈ 0.69 and 〈*R*(*t*)〉 ≈ 0.21 for strongly and weakly synchronized regimes of STN neurons illustrated in Fig 2A, 2B, 2C and 2D, respectively.

### Feedback stimulation protocols

We consider the two above cases of strongly and weakly synchronized regimes and show how the external stimulation based on delayed feedback can suppress the neuronal synchronization. We investigate the impact of two different feedback stimulation techniques counteracting neuronal synchronization. The first stimulation protocol is a linear delayed feedback (LDF) [55, 56]. To calculate the LDF stimulation signal, the LFP of synchronized STN neurons is measured. We model the LFP as an averaged synaptic activity of neurons $LFP\left(t\right)=N^{-1}\sum_{j=1}^{N}s_{j}$ [83], where *s*_*j*_(*t*) are the synaptic variables Eq (4) of STN neurons, see also papers [84, 85] for a more sophisticated approach. The measured LFP is on-line filtered by applying a linear damped oscillator

$$\begin{array}{c} ü+\alpha_{d}\dot{u}+\omega^{2}u=k_{f}LFP\left(t\right). \end{array}$$ (5)

Parameter *ω* approximates the frequency of the LFP oscillations *ω* = 2*π*/*T*, where *T* is the mean period of LFP. For the strongly synchronized state illustrated in Fig 2A and 2B, *T* ≈ 110 ms. In a real application the mean period *T* for filtering can be selected, for example, based on the central frequency of the pronounced spectral peak in the *β*-band of the measured LFP observed in Parkinson’s disease [52, 53, 78, 86, 87]. As the output signal of Eq (5), that is the filtered LFP, we use the variable $x\left(t\right)=\dot{u}$, which has a zero phase shift with respect to the original LFP signal [60]. The damping and scaling coefficients in Eq (5) were chosen *α_d_* = *k*_f_ = 0.008 which preserves the amplitude of the input raw LFP signal, see S1 Text for a parameter optimization approach for filtering. The filtered LFP signal is illustrated in Fig 2B and 2D by red curves for strongly and weakly synchronized regimes of STN neurons, respectively.

The stimulation signal *I*_*stim*_ in Eq (1) of the differential LDF is then calculated as [55, 56]:

$$I_{\text{stim}}=K(x(t-\tau )-x(t)),$$ (6)

where *K* is the parameter of the stimulation intensity, and *τ* is the stimulation delay.

Another control method considered in this study is based on nonlinear delayed feedback (NDF) suggested in Refs. [57, 61] for the control of pathological neuronal synchronization. To construct the stimulation signal, we represent the measured mean field of the synchronized neuronal population in the form of an analytic complex signal *Z*(*t*) = *x*(*t*) + i*y*(*t*), where the variable *x*(*t*) is the filtered LFP signal obtained with the help of Eq (5) as for the case of LDF stimulation, and the corresponding *y*(*t*) signal can be calculated from *x*(*t*) by means of Hilbert transform [4]. In a simple realization, which we use in this study, *y*(*t*) can be approximated by the time-shifted filtered LFP, *y*(*t*) = *x*(*t* − *T*/4), where *T* is the mean period of LFP. The stimulation signal of the NDF reads *S*(*t*) = *KZ*^2^(*t*)Z*(*t* − *τ*), where the asterisk denotes the complex conjugacy. In our case we consider only the real part of *S*(*t*) as the stimulation signal

$$\begin{array}{c} I_{\text{stim}}=Kx\left(t-\tau \right)\left(x^{2}\left(t\right)-y^{2}\left(t\right)\right)+2Kx\left(t\right)y\left(t\right)y\left(t-\tau \right), \end{array}$$ (7)

where, as before, *K* is the stimulation intensity, and *τ* is the stimulation delay.

## Results

### Feedback stimulation with smooth signals

In this section we address the desynchronizing impact of the LDF and NDF stimulations with the stimulation signals Eqs (6) and (7), respectively. The stimulation signals are smooth and slowly oscillating since they are calculated from the filtered and, thus, smooth LFP of STN neurons. The stimulation is administered to STN neurons only, and the GPe neurons are not stimulated. We compare the effect of the stimulation for two conditions of strongly and weakly coupled neurons, where the neuronal population exhibits strongly and weakly synchronized intrinsic dynamics, respectively, as illustrated in Fig 2.

#### Linear delayed feedback

**Fig 3:**
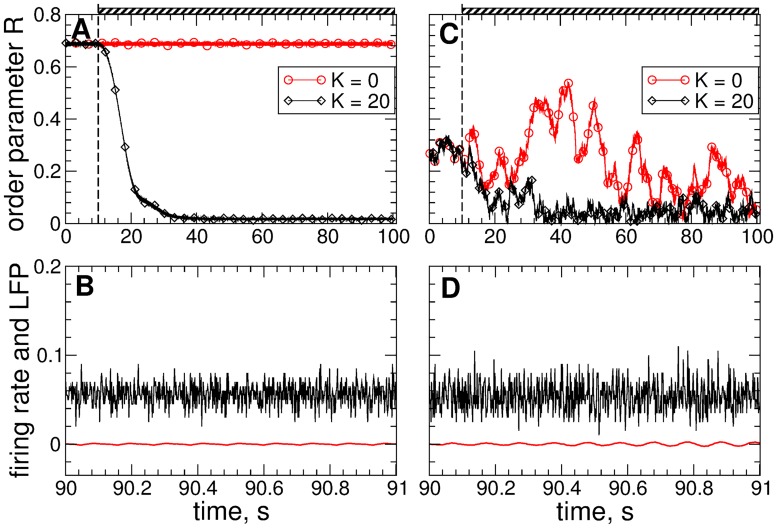
Suppression of synchronization in the neuronal ensemble Eqs (1)–(3) by the LDF stimulation Eq (6). Time courses of the order parameter *R* of STN neurons for the stimulation intensity *K* = 0 (stimulation-free case) and *K* = 20 are shown in plots (A) and (C) for strongly coupled (*g*_G→S_ = 1.7 nS/*μ*m^2^) and weakly coupled (*g*_G→S_ = 1.28 nS/*μ*m^2^) regimes, respectively. The stimulation epochs are indicated by hatched bars on the top of the plots. In plots (B) and (D) the corresponding firing rates (black curves) and filtered LFPs (red curves) of the stimulated STN neurons are depicted for the strongly and weakly coupled cases, respectively. Parameter *τ* = 95 ms.

The desynchronizing impact of the differential LDF Eq (6) on the STN neurons is illustrated by the example in Fig 3. The order parameter *R*(*t*) of the STN without stimulation (stimulation intensity *K* = 0) saturates at 〈*R*〉 ≈ 0.69 for the case of strong coupling [Fig 3A, red circles] and fluctuates around 〈*R*〉 ≈ 0.21 for the case of weak coupling [Fig 3C, red circles]. In the latter case the STN neurons exhibit weak and intermittent synchronization, see Fig 2C and 2D. For the fixed stimulation delay *τ* = 95 ms and stimulation intensity *K* = 20, the differential LDF can significantly suppress the neuronal synchronization in the strongly coupled regime, where the values of the order parameter are reduced to 〈*R*〉 ≈ 0.02 (averaged during the last 20 s) [Fig 3A, black diamonds]. The stimulation-induced desynchronized dynamics is also reflected by the STN firing rate and behavior of LFP, where no rhythmic oscillations are observed any longer, see Fig 3B and compare to Fig 2B. Also for the case of weak coupling and weak and intermittent initial synchronization the differential LDF is still effective in counteracting neuronal synchronization. For the same stimulation parameters, the values of the order parameter are reduced to 〈*R*〉 ≈ 0.05 [Fig 3C, black diamonds], and the low-amplitude oscillations of the firing rate and LFP are further suppressed, see Fig 3D and compare to Fig 2D. The time course of desynchronization is monotonous in the case of strong coupling, where the feedback signal is regular [Fig 3A], while for weak coupling, where the feedback signal is contaminated by statistically relevant fluctuations, the time course of desynchronization displays fluctuations (and even epochs of slight increase of synchrony) as well [Fig 3C].

**Fig 4:**
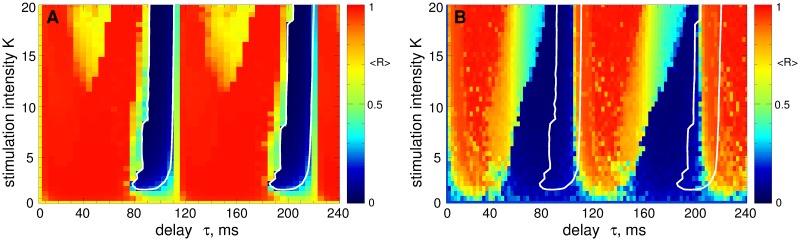
Impact of the LDF stimulation Eq (6) on the neuronal ensemble Eqs (1)–(3). The time-averaged order parameter 〈*R*(*t*)〉 of the stimulated STN neurons is depicted in color ranging from 0 (blue) to 1 (red) versus feedback delay *τ* and stimulation intensity *K* for (A) strongly coupled regime (*g*_G→S_ = 1.7 nS/*μ*m^2^) and (B) weakly coupled regime (*g*_G→S_ = 1.28 nS/*μ*m^2^). The white curves in (A) delimit the parameter domains of effective desynchronization, where 〈*R*〉 < 0.25. The same curves are also depicted in plot (B) for comparison.

The LDF stimulation can have different impact on the collective dynamics of the stimulated neurons depending of the stimulation parameters. It is known that in the parameter plane of the stimulation delay and intensity (*τ*, *K*), there are regions of desynchronization complemented by domains, where synchronization is enhanced by stimulation [55, 56, 61]. This property is illustrated for the considered model in Fig 4. For example, for the strongly coupled regime, the LDF can induce a nearly perfect desynchronization in the blue parameter islands depicted in Fig 4A, where the order parameter practically vanishes, see also Fig 3A. In the red parameter domain in Fig 4A, on the other hand, the LDF stimulation forces the neurons to synchronize, and the order parameter closely approaches 1. Therefore, for a successful suppression of the neuronal synchronization the stimulation parameters have to be calibrated to the desynchronization regions. The situation is similar for the case, when the LDF stimulation is applied to weakly coupled and weakly synchronized STN neurons, which is illustrated in Fig 4B. The desynchronization regions are however larger for this regime, in particular, for a weak to moderate stimulation intensity.

It was also shown [55, 56] that the properties of the desynchronization regions, e.g., size, location, form, etc., depend on the properties and parameters of the stimulated system, such as oscillation frequency, extent of synchronization, coupling, etc. For example, changing the oscillation frequency of the stimulated neurons may shift the desynchronization regions in the (*τ*, *K*)-parameter plane with respect to the delay parameter *τ*. This effect is observed in Fig 4B for weakly coupled regime. Indeed, the period *T* of the LFP oscillations in the weakly coupled regime is approximately *T* ≈ 100 ms [Fig 2D] which is smaller than that for the strongly coupled neurons, where *T* ≈ 110 ms [Fig 2B]. This causes a shift of the desynchronization regions toward smaller values of the stimulation delay *τ*, as shown in Fig 4B (compare blue regions with those in Fig 4A bounded by white curves). This effect is more pronounced for large values of *τ*. It is also known that large values of stimulation delay and stimulation intensity are not favorable for a desynchronizing effect of LDF [55, 56, 61]. Therefore, for the optimal range of the stimulation parameters (small *τ* and *K*), the desynchronization induced by the LDF stimulation in the strongly synchronized neuronal population is preserved if the intrinsic neuronal dynamics changes to a weakly synchronized regime, except for a narrow boundary region, which has to be avoided also for the strongly synchronized case. Note, the authors of Ref. [73] considered the fixed stimulation delay *τ* = 50 ms which does not belong to the desynchronization regions [Fig 4A, blue domains] and, therefore, is not representative for the desynchronization effect of the LDF stimulation for the considered model.

#### Linear delayed feedback with adaptive parameters

For the case, when the stimulation parameters of the LDF are sub-optimally selected or shifted out of the desynchronization regions [Fig 4] because of the fluctuation of system properties (frequency, synchronization strength, etc.), they have to be recalibrated. This can be done automatically as suggested by Montaseri *et al*. [64], where the stimulation parameters can be adapted depending on the extent of the stimulation-induced desynchronization. The proposed calibration algorithm adjusts the stimulation parameters based on, for example, the amplitude of the LFP. Following Ref. [64], together with Eq (5) we consider an additional variable *w*(*t*):

$$\begin{array}{c} \dot{w}=\frac{1}{\mu }\left(\dot{u}-w\right), \end{array}$$ (8)

where *μ* is some large coefficient such that *μω* ≫ 1, for instance, *μ* = 2000, and Eq (8) acts as an integrator. Since the variable $x\left(t\right)=\dot{u}$ is the filtered LFP, its amplitude can be found as $X=\sqrt{x^{2}+\mu^{2}\omega^{2}w^{2}}$. The rule of the automatic parameter calibration adapted from [64] reads

$$\begin{array}{c} \begin{array}{ccc} \dot{K} & = & b_{1}X\left(1+\tanh\left[a_{1}\left(X-X_{1}\right)\right]\right),\\ \dot{\tau } & = & b_{2}X\left(1+\tanh\left[a_{1}\left(X-X_{1}\right)\right]\right)\left(1+\tanh\left[a_{2}\left(X-X_{2}\right)\right]\right), \end{array} \end{array}$$ (9)

where parameters *X*_1_ = 0.005 and *X*_2_ = 0.028 define the cutoff thresholds for the growth of the stimulation parameters *K* and *τ*, respectively, *a*_1_ = 4000 and *a*_2_ = 500 determine the widths of the cutoffs, and *b*_1_ = 7.5 ⋅ 10^−6^ and *b*_2_ = 5 ⋅ 10^−4^ govern the speed of the parameter variation.

**Fig 5:**
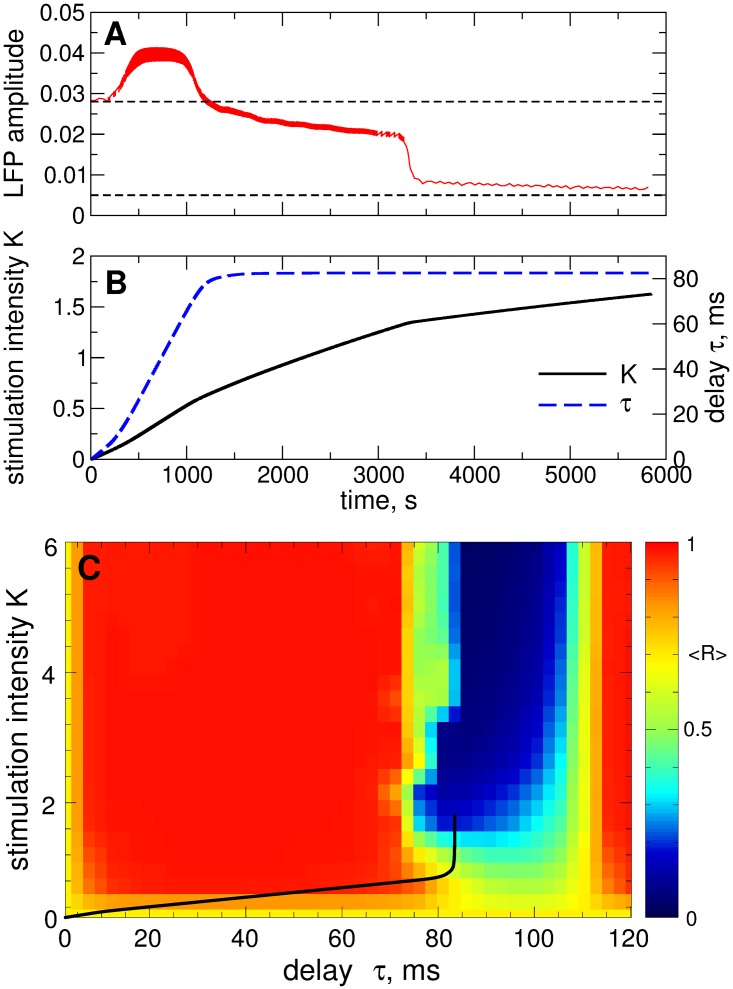
Automatic selection of the stimulation parameters *τ* and *K* for the desynchronizing LDF stimulation. (A) Time course of the amplitude of the LFP and (B) the evolution of the stimulation intensity *K* and the feedback delay *τ* (as indicated in the legend) during the parameter calibration process governed by Eq (9). In plot (A) the upper and lower horizontal dashed lines indicate the cutoff thresholds of the growth of the stimulation parameters *τ* and *K*, respectively, see parameters *X*_2_ and *X*_1_ in Eq (9). In plot (B) the scale for parameter *τ* is shown on the right vertical axis. (C) The corresponding pathway of the (*τ*, *K*)-parameter point is depicted by the black curve in the (*τ*, *K*)-parameter plane, where a part of Fig 4A is enlarged. The values of the time-averaged order parameter 〈*R*(*t*)〉 of the stimulated STN neurons are encoded by color. Parameter *g*_G→S_ = 1.7 nS/*μ*m^2^.

The performance of the parameter calibration algorithm Eqs (8) and (9) is illustrated in Fig 5. Starting at (*τ*(0), *K*(0)) = (0, 0), both parameters increase [Fig 5B] until the LFP amplitude [Fig 5A] falls below the cutoff threshold for the delay (parameter *X*_2_ in Eq (9), upper dashed line in Fig 5A). Then the growth of *τ* is slowed down, whereas the stimulation intensity *K* continues to increase and saturates when the LFP amplitude approaches the cutoff threshold for *K* (parameter *X*_1_ in Eq (9), lower dashed line in Fig 5A). At the end of the parameter calibration process the stimulation parameters reach the desynchronization region [Fig 5C], and the values of the order parameter are successfully lowered from 〈*R*〉 ≈ 0.69 at the initial state to 〈*R*〉 ≈ 0.16. The illustrated algorithm can also be used in the case when the parameters of the stimulated neuronal population are non-stationary, see Ref. [64] for details.

#### Nonlinear delayed feedback

**Fig 6:**
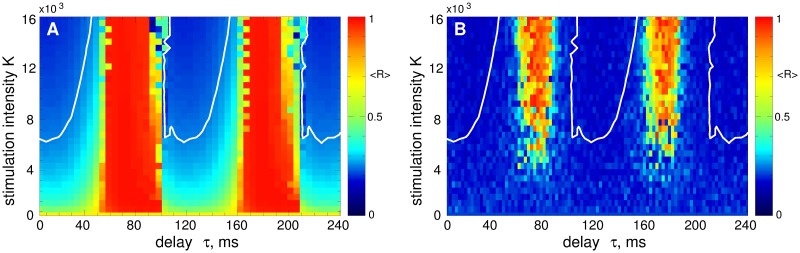
Impact of the NDF stimulation Eq (7) on the neuronal ensemble Eqs (1)–(3). The time-averaged order parameter 〈*R*(*t*)〉 of the stimulated STN neurons is depicted in color ranging from 0 (blue) to 1 (red) versus feedback delay *τ* and stimulation intensity *K* for (A) strongly coupled regime (*g*_G→S_ = 1.7 nS/*μ*m^2^) and (B) weakly coupled regime (*g*_G→S_ = 1.28 nS/*μ*m^2^). The white curves in (A) bound the parameter domains of effective desynchronization, where 〈*R*〉 < 0.25. The same curves are also depicted in plot (B) for comparison.

In this section we illustrate the desynchronizing effect of the NDF stimulation Eq (7) administered to the considered model of STN neurons Eqs (1)–(3). In Fig 6 the time-averaged order parameter 〈*R*〉 is depicted by color versus the parameters of the stimulation delay *τ* and stimulation intensity *K* for two cases of strong coupling [Fig 6A] and weak coupling [Fig 6B], which correspond to strongly and weakly synchronized STN neurons without stimulation, respectively, see Fig 2. Starting from the mentioned initial states of the STN, the NDF stimulation can effectively desynchronize the stimulated neurons with stimulation parameters selected from the desynchronization parameter regions showing up in blue color in Fig 6. The desynchronization regions for the two considered regimes are located at nearly the same places in the (*τ*, *K*)-parameter plane although the stimulation-free dynamics of the strongly and weakly coupled neurons differ from each other, e.g., by oscillation frequency and extent of synchronization. The NDF desynchronization method is thus less sensitive to the variation of these properties of the neuronal population: If strongly synchronized neurons can be desynchronized by the NDF stimulation, the desynchronization is also induced by stimulation with the same parameters in the case of weakly synchronized neurons (compare blue regions bounded by the white curves in Fig 6A and 6B).

#### Efficacy of linear and nonlinear delayed feedbacks

The desynchronization mechanism of NDF differs from that of LDF. The theory of LDF says that the desynchronization regions are bounded by bifurcation curves, where the amplitude of the ensemble mean field vanishes, which corresponds to a state of perfect desynchronization, see Refs. [55, 56, 88]. The NDF stimulation, on the other hand, does not cause any bifurcation, but the amplitude of the mean field is gradually suppressed and decays ∼*K*^−1/2^ as the stimulation intensity *K* increases [57, 61]. For a network of two interacting populations, where only one population is measured and stimulated, the mean field of the stimulated neurons decays ∼*K*^−1/3^ as *K* grows [63]. The latter case corresponds to the stimulation setup considered in this study, where only STN neurons from the STN-GPe network are recorded and stimulated.

**Fig 7:**
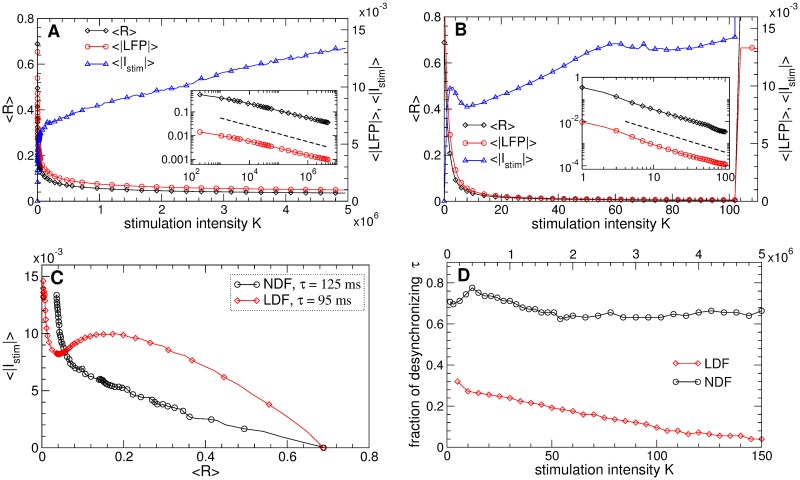
Efficacy of synchronization control in the neuronal ensemble Eqs (1)–(3) by the NDF and LDF stimulations. (A), (B) The time-averaged order parameter 〈*R*〉, the absolute value of the filtered LFP 〈|*LFP*|〉, and the absolute value of the stimulation signal 〈|*I*_*stim*_|〉 are depicted versus *K* for (A) NDF stimulation Eq (7) and (B) LDF stimulation Eq (6) as indicated in the legends. The scaling for the latter two quantities is given on the right vertical axes. In the inserts, 〈*R*〉 and 〈|*LFP*|〉 are plotted on a log-log scale, where the dashed lines have the slopes (A) −1/3 and (B) −1 and are given for comparison. (C) Amount of the administered stimulation as given by 〈|*I*_*stim*_|〉 versus the reached extent of desynchronization as given by 〈*R*〉 for NDF and LDF stimulations as indicated in the legend. (D) Fraction of parameter *τ* ∈ (0, 250) ms values, where the LDF or NDF stimulation (as indicated in the legend) induces a desynchronization of STN neurons with 〈*R*〉 < 0.35 versus the stimulation intensity *K*. Parameters *g*_G→S_ = 1.7 nS/*μ*m^2^, (A) *τ* = 125 ms and (B) *τ* = 95 ms.

The behavior of the time-averaged order parameter 〈*R*〉, the absolute value of the filtered LFP 〈|*LFP*|〉, and the absolute value of the stimulation signal 〈|*I*_*stim*_|〉 from Eq (7) of the NDF stimulation is shown in Fig 7A versus the stimulation intensity *K*. As expected, order parameter and LFP decay as *K* grows according to a power law ∼*K*^*γ*^, see the insert in Fig 7A. For the considered STN population of *N* = 200 neurons, the exponent *γ* slightly deviates from the predicted −1/3 [63], where the direct numerical fit gives *γ* ≈ −0.3, which may be related to the finite-size effect [89]. We however evaluate the properties of the feedback stimulation for the considered finite-size neuronal population. Because of such a value of *γ*, the amplitude of the stimulation signal 〈|*I*_*stim*_|〉 slowly increases when *K* grows [Fig 7A, blue triangles], cf. Ref. [63]. Nevertheless, even for large values of the stimulation intensity *K*, where the order parameter can reach 〈*R*〉 ≈ 0.035 indicating an about 20-fold suppression of synchronization, the amplitude of the stimulation signal 〈|*I*_*stim*_|〉 is still smaller than the amplitude of the LFP 〈|*LFP*|〉 ≈ 0.018 of the synchronized STN neurons without stimulation, see Fig 7A. Therefore, the NDF technique can effectively suppress the neuronal synchronization by a weak stimulation as compared, for instance, to HF DBS.

We evaluate the dynamics of the quantities 〈*R*〉, 〈|*LFP*|〉, and 〈|*I*_*stim*_|〉 also for the LDF stimulation Eq (6), which are depicted in Fig 7B versus *K*. The order parameter and the amplitude of LFP decay as *K* increases much faster than for the NDF stimulation, and the mentioned level of desynchronization 〈*R*〉 ≈ 0.035 can be reached already for *K* = 10 and ranges to 〈*R*〉 ≈ 0.0035 for *K* = 100. However, the amplitude of the stimulation signal 〈|*I*_*stim*_|〉 also increases as the stimulation intensity grows [Fig 7B, blue triangles]. To compare the efficacy of the LDF and NDF stimulations we plot in Fig 7C the amount of the administered stimulation as given by the amplitude of the stimulation signal 〈|*I*_*stim*_|〉 from Eqs (6) and (7), respectively, versus the reached extent of desynchronization as given by values of 〈*R*〉 in Fig 7A and 7B. For a range of the order parameter *R* > 0.055 the NDF stimulation is more efficient in inducing desynchronization than the LDF stimulation for a given ensemble size: To reach the same extent of desynchronization the NDF requires a smaller amount of the stimulation. However, if we need a very strong desynchronization, then the LDF stimulation is more appropriate.

Stronger desynchronization can be obtained by the LDF stimulation for a larger stimulation intensity *K*, which also results in a large amplitude of the stimulation signal 〈|*I*_*stim*_|〉, see Fig 7B. Furthermore, for too large *K* the desynchronization regions [Fig 4A, blue domains] shrink, and the LDF stimulation becomes ineffective in inducing desynchronization, see Refs. [55, 56, 61]. For example, for *τ* = 95 ms the LDF stimulation can boost synchronization instead of suppressing it for *K* > 102 [Fig 7B]. In Fig 7D we plot the fraction of the parameter *τ* values, where the LDF and NDF stimulations can suppress synchronization with 〈*R*〉 < 0.35, i.e., at least twice as compared to the stimulation-free regime, where 〈*R*〉 ≈ 0.69. The desynchronization regions of the LDF stimulation can occupy up to 35% of the (*τ*, *K*)-parameter plane for small *K*, whereas they can be reduced to only 4% of the parameter volume for large *K* [Fig 7D, red diamonds]. For the NDF stimulation, the desynchronization regions occupy more than 65% of the (*τ*, *K*)-parameter plane, and this property seems to be independent of the parameter of the stimulation intensity *K* [Fig 7D, black circles]. Therefore, large values of parameter *K* are not desirable for the LDF stimulation, whereas they can be beneficial for the NDF stimulation.

### Pulsatile feedback stimulation protocols

The stimulation signals Eqs (6) and (7) of the LDF and NDF, respectively, are derived from the filtered LFP. The latter is a smooth signal and oscillates at the frequency ≈ 9−10 Hz for the considered model and parameters [Fig 2], which results in smooth stimulation signals Eqs (6) and (7) oscillating at these frequencies. When administered to the stimulated neurons as electrical stimulation, e.g., via a deeply implanted electrode, the neurons receive a stimulation current of the same polarity during a half of the oscillation period, which lasts several tens of milliseconds. Such a long stimulation phase of the same polarity may cause an irreversible and possibly impairing charge deposit in the vicinity of the electrode, which may violate safety requirements of the electrical stimulation of the neuronal tissue and lead to its damage [16, 69, 70].

The stimulation signal of the standard HF DBS consists of a pulse train of biphasic electrical pulses administered at high frequencies above 100 Hz, often at 130 Hz [22]. The cathodic and anodic phases of each pulse should deliver the same charge of opposite polarity providing a charge-balanced stimulation with zero net charge injection into the stimulated tissue, which is a necessary requirement to avoid a tissue damage [16, 69, 70, 90].

**Fig 8:**
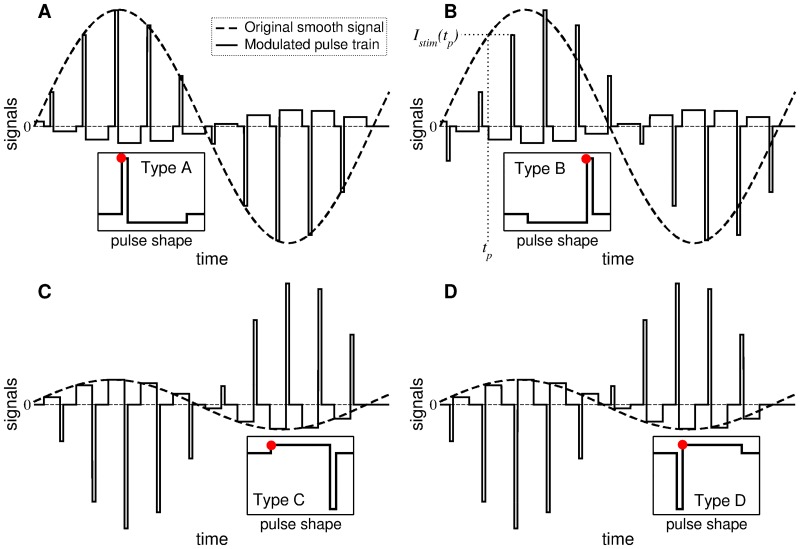
Stimulation signals of pulsatile delayed feedback. The amplitude of the high-frequency pulse train of the charge-balanced asymmetric biphasic pulses is modulated by a slowly oscillating smooth signal of LDF Eq (6) or NDF Eq (7). The corresponding four shapes of single pulses are schematically depicted in the inserts. In plot (B), examples of the pulse onset at *t* = *t*_*p*_ and the corresponding value of the smooth feedback signal *I*_stim_(*t_p_*) are indicated by dotted lines, where the latter value is assigned in this case to the second, short phase of the biphasic pulse. Red dots in the inserts indicate the phases of the pulses, whose amplitude is calculated as *I*_stim_(*t_p_*).

Combining both approaches, we construct a stimulation signal which inherits the advantages of the charge-balanced property of the HF DBS signal and the desynchronizing impact of the delayed feedback stimulation. In such a way, the amplitude of the high-frequency pulse train is modulated by the slowly oscillating smooth signal of the LDF Eq (6) or NDF Eq (7), which is schematically shown in Fig 8. We consider four types of the asymmetric biphasic charge-balanced pulses illustrated in the inserts in Fig 8. The typical waveform of the biphasic charge-balanced stimulation pulses used for the standard HF DBS consists of a short first pulse (1st phase) of duration 60 to 450 *μ*sec [22] followed by a charge-balancing 2nd phase of opposite polarity such that the total charge of the biphasic pulse is zero [68]. We consider two cases of the anodic 1st phase [Fig 8A] and the cathodic 1st phase [Fig 8D]. The latter pulse shape is a standard pulse waveform widely used for HF DBS [68]. In our numerical simulations we also consider additional pulse shapes illustrated in Fig 8B and 8C, where the 1st and the 2nd phases of the stimulation pulses in Fig 8A and 8D are exchanged with each other, respectively, and the charge-balancing phase advances the short stimulation pulse. We consider the frequency of the pulse train 130 Hz (the inter-pulse interval 1000/130 ≈ 7.69 ms). The width of the short pulse (the 1st phase in Fig 8A and 8D and the 2nd phase in Fig 8B and 8C) is taken *PW* = 0.5 ms or 0.2 ms, and relates to the duration of its long counterpart as 1: 10, which is found to be energy efficient [25]. Potentially, charge-balanced waveforms could be incorporated in other feedback methods, such as PRC based ones [67].

For a given smooth signal *I*_stim_(*t*) of the delayed feedback calculated according to Eqs (6) or (7) [Fig 8, dashed curves], the amplitude of a stimulation pulse is calculated at the time *t* = *t*_*p*_ of the pulse onset as *I*_stim_(*t_p_*). Then, depending on the pulse shape, see insets in Fig 8, this value is assigned either to the short phase of the biphasic pulse as in Fig 8A and 8B, or to the long phase of the pulse as in Fig 8C and 8D, as indicated by red dots in the inserts. The amplitude of the other counterpart of the pulse is obtained from the charge-balancing property such that the square delineated by the biphasic pulse is zero. Examples of the values *t*_*p*_ (pulse onset) and *I*_stim_(*t_p_*) are indicated in Fig 8B by dotted lines, where the value *I*_stim_(*t_p_*) is assigned in this case to the short (second) phase of the pulse.

#### Pulsatile linear delayed feedback

**Fig 9:**
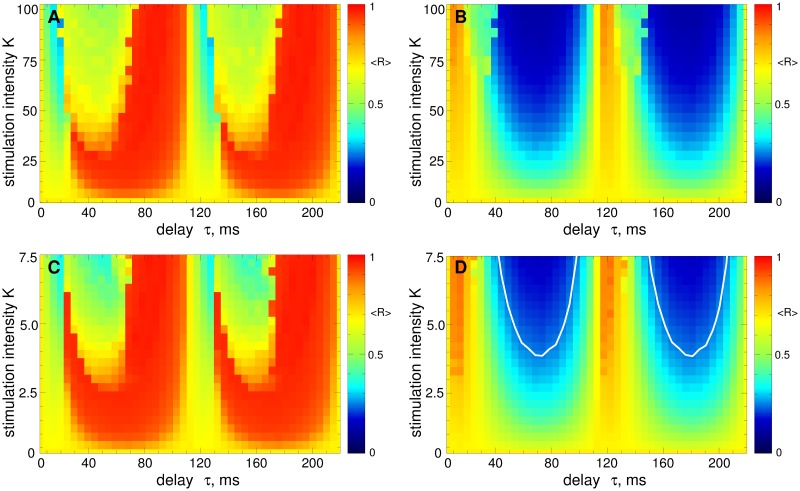
Impact of the pulsatile LDF stimulation on the neuronal ensemble Eqs (1)–(3). The time-averaged order parameter 〈*R*(*t*)〉 of the stimulated STN neurons is depicted in color ranging from 0 (blue) to 1 (red) versus the feedback delay *τ* and the stimulation intensity *K* for a strongly coupled regime (*g*_G→S_ = 1.7 nS/*μ*m^2^). The plots (A)—(D) are obtained for the stimulation signals in Fig 8(A)–8(D), respectively, where the amplitude of the pulse train in modulated by the LDF signal Eq (6). The white curves in (D) delimit the parameter domains of effective desynchronization, where 〈*R*〉 < 0.25; a similar large domain of effective desynchronization can be seen on panel (B). Pulse width *PW* = 0.5 ms.

We illustrate the impact of the pulsatile LDF stimulation on the STN neurons Eqs (1)–(3) in Fig 9 for the case of strong coupling *g*_G→S_ = 1.7 nS/*μ*m^2^. Starting from the initially synchronized regime [Fig 2A and 2B] the stimulation of STN neurons with the pulse train modulated by the LDF signal Eq (6) can suppress the neuronal synchronization. The efficacy of the stimulation depends on the type of the charge-balanced biphasic pulses. For the pulses of type A or C [Fig 8A and 8C] the pulsatile LDF stimulation is hardly effective [Fig 9A and 9C]. On the other hand, for the pulses of type B or D [Fig 8B and 8D], the pulsatile LDF can pronouncedly desynchronize the stimulated neurons in large parameter regions [Fig 9B and 9D]. The size of the desynchronization regions approaches that for the NDF stimulation with smooth stimulation signal, compare the size of blue regions in Fig 9B and 9D with that in Fig 6A and in Fig 4A.

#### Pulsatile nonlinear delayed feedback

**Fig 10:**
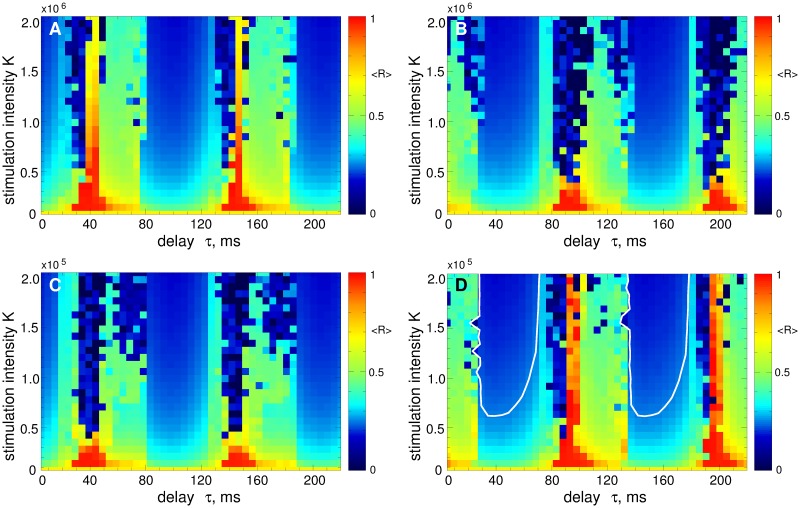
Impact of the pulsatile NDF stimulation on the neuronal ensemble Eqs (1)–(3). The time-averaged order parameter 〈*R*(*t*)〉 of the stimulated STN neurons is depicted in color ranging from 0 (blue) to 1 (red) versus the feedback delay *τ* and the stimulation intensity *K* for strongly coupled regime (*g*_G→S_ = 1.7 nS/*μ*m^2^). The plots (A)—(D) are obtained for the stimulation signals in Fig 8(A)–8(D), respectively, where the amplitude of the pulse train in modulated by the NDF signal Eq (7). The white curves in (D) bound the largest continuous parameter domains of effective desynchronization, where 〈*R*〉 < 0.25. Pulse width *PW* = 0.5 ms.

The impact of the pulsatile NDF stimulation on the STN neurons Eqs (1)–(3) is illustrated in Fig 10 for the case of strong coupling *g*_G→S_ = 1.7 nS/*μ*m^2^. When the pulse train is modulated by the NDF signal Eq (7), the neurons can effectively be desynchronized irrespectively of the considered type of the the charge-balanced biphasic pulses. The desynchronization regions are very similar in size (although they can be shifted with respect to parameter *τ*) in all four plots in Fig 10 (blue domains), which indicates that the pulsatile NDF stimulation is little sensitive to the waveform of the stimulation pulses.

#### Desynchronization of weakly coupled regime

**Fig 11:**
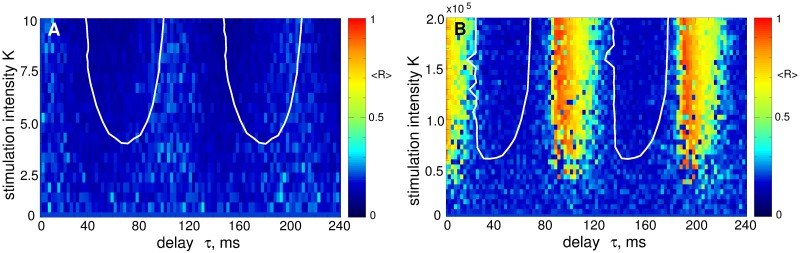
Impact of the pulsatile delayed feedback stimulation on the weakly coupled neuronal ensemble Eqs (1)–(3). The time-averaged order parameter 〈*R*(*t*)〉 of the stimulated STN neurons is depicted in color ranging from 0 (blue) to 1 (red) versus the feedback delay *τ* and the stimulation intensity *K* for a weakly coupled regime (*g*_G→S_ = 1.28 nS/*μ*m^2^). The stimulation signal consists of a pulse train of the charge-balanced biphasic pulses of type D [Fig 8D] modulated by (A) the LDF signal Eq (6) and (B) NDF signal Eq (7). The white curves in plots (A) and (B) are the boundaries of the largest continuous desynchronization regions from Figs 9D and 10D, respectively, and given for comparison. Pulse width *PW* = 0.5 ms.

To address the problem mentioned by Dovzhenok *et al*. [73] that the desynchronizing feedback stimulation may boost synchronization when administered to initially intermittently and weakly synchronized regimes, we apply the pulsatile LDF and NDF stimulations to the STN neurons in the weakly coupled regime for *g*_G→S_ = 1.28 nS/*μ*m^2^ [Fig 2C and 2D] for the same stimulation parameters as in Figs 9 and 10. We consider the pulses of type D [Fig 8D] widely used for the standard HF DBS [68]. The results of the stimulation are shown in Fig 11A and 11B for the pulsatile LDF and NDF stimulations, respectively. Both pulsatile LDF and NDF stimulations are robust with respect to the variation of the extent of synchronization in the neuronal population. Indeed, for the parameters from the desynchronization regions delimited by the white curves in Fig 9D for LDF stimulation of initially strongly coupled and strongly synchronized neurons, the pulsatile LDF stimulation does not cause any enhancement of synchronization also for the ensembles of weakly coupled and weakly synchronized neurons, see Fig 11A, where the boundaries of desynchronization regions from Fig 9D are depicted by white curves for comparison. Also for the pulsatile NDF stimulation, the largest continuous desynchronization regions obtained for initially strongly coupled and strongly synchronized neurons [Fig 10D] nearly perfectly fit to the desynchronization regions for initially weakly coupled and weakly synchronized neurons, see Fig 11B (white curves). Our results cannot confirm the conclusions of Dovzhenok *et al*. [73] for the pulsatile LDF and NDF stimulations. For an appropriate selection of the stimulation parameters causing a pronounced desynchronization of initially strongly synchronized neurons, the stimulation by pulsatile LDF and NDF is not expected to cause any problem when the neuronal population runs into a regime of weak or intermittent synchronization.

#### Efficacy of pusatile linear and nonlinear delayed feedbacks

**Fig 12:**
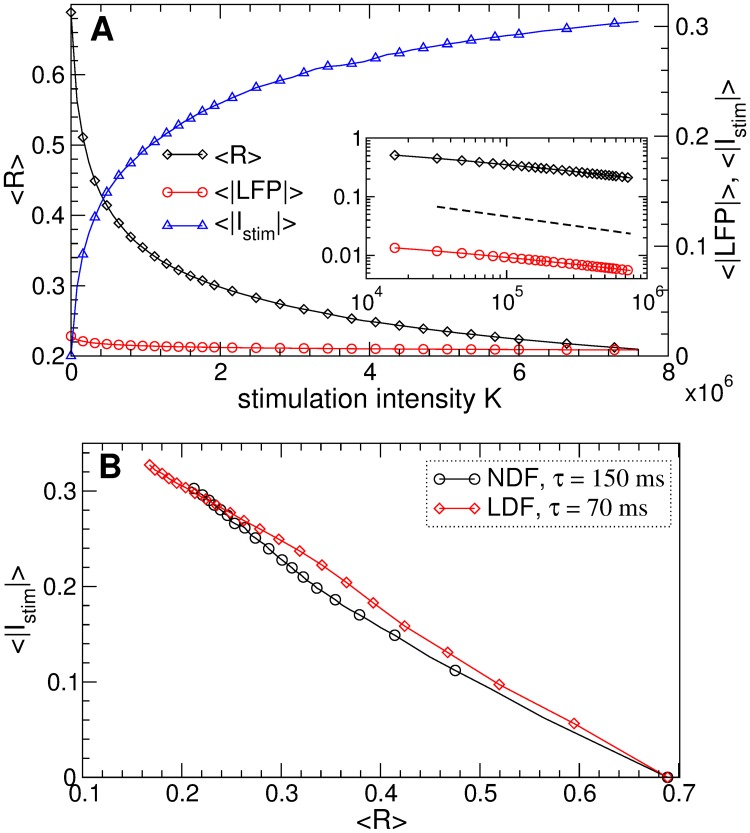
Efficacy of synchronization control in the neuronal ensemble Eqs (1)–(3) by the pulsatile LDF and NDF stimulations. (A) The time-averaged order parameter 〈*R*〉, the absolute value of the filtered LFP 〈|*LFP*|〉, and the absolute value of the stimulation signal 〈|*I*_*stim*_|〉 are depicted versus *K* for the stimulation by pulse train of the charge-balanced biphasic pulses of type D [Fig 8D] modulated by the NDF signal Eq (7). The scaling for 〈|*LFP*|〉 and 〈|*I*_*stim*_|〉 is given on the right vertical axis. In the insert, 〈*R*〉 and 〈|*LFP*|〉 are plotted in the log-log scale, where the dashed line has the slope−1/3 and is given for comparison. Parameter of delay *τ* = 150 ms. (B) Amount of the administered stimulation as given by 〈|*I*_*stim*_|〉 versus the reached extent of desynchronization as given by 〈*R*〉 for the pulsatile NDF and LDF stimulations as indicated in the legend. 〈|*I*_*stim*_|〉 for LDF and NDF is calculated from the smooth signals Eqs (6) and (7), respectively, modulating the stimulation pulses as shown in Fig 8. Parameter *g*_G→S_ = 1.7 nS/*μ*m^2^, and pulse width *PW* = 0.2 ms.

The suggested pulsatile stimulation by LDF or NDF are also robust with respect to a variation of the pulse parameters. To illustrate this we consider a shorter pulse width *PW* = 0.2 ms. Also with such pulses of type D [Fig 8D] the pulsatile NDF stimulation can effectively desynchronize the STN neurons, where the quality of desynchronization is improved (the order parameter 〈*R*〉 and the amplitude of the LFP 〈|*LFP*|〉 decay) as the stimulation intensity *K* increases, see Fig 12A. As for the smooth NDF [Fig 7A], the decay of 〈*R*〉 and 〈|*LFP*|〉 obeys a power law ∼*K*^*γ*^, see the insert in Fig 12A. Since the considered neuronal model has a finite size, the numerically obtained exponent *γ* slightly deviates from the theoretically predicted *γ* = −1/3 [63], which leads to a slow increase of the amplitude 〈|*I*_*stim*_|〉 of the stimulation signal [Fig 12A, blue triangles]. Hence, for a better desynchronization the neurons have to be stimulated somewhat stronger. The same holds for the pulsatile LDF stimulation, and we illustrate the efficacy of both pulsatile feedback methods in Fig 12B, where the amount of the administered stimulation as given by 〈|*I*_*stim*_|〉 is plotted versus the reached extent of desynchronization as given by 〈*R*〉. If compared to the case of smooth stimulation signals [Fig 7C], the pulsatile stimulation protocol diminishes the difference in the efficacy of NDF and LDF, where both methods require approximately the same amount of the stimulation to reach the given level of desynchronization. A similar conclusion can be drawn with respect to the size of desynchronization regions, except for the sensitivity of the pulsatile LDF and NDF to the shape of individual pulses, see Figs 9 and 10.

#### Robustness with respect to slowly varying parameters

**Fig 13:**
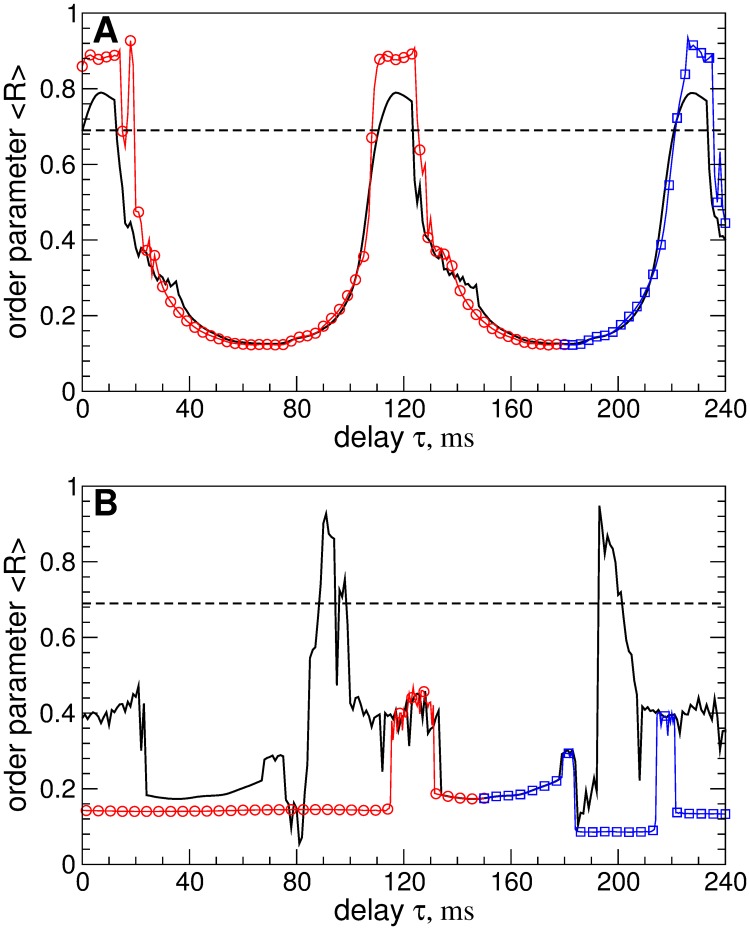
Synchronization control in the neuronal ensemble Eqs (1)–(3) by the pulsatile LDF and NDF stimulations for slowly varying stimulation delay *τ*. The time-averaged order parameter 〈*R*〉 of the stimulated STN neurons is plotted versus *τ* for (A) pulsatile LDF and (B) pulsatile NDF stimulation by pulse train of the charge-balanced biphasic pulses of type D [Fig 8D]. The black solid curves depict the stimulation-induced values of 〈*R*〉 from Figs 9D and 10D, where the stimulation is administered to initially synchronized neurons, see Figs 2A, 2B and 3A. The red circles and blue squares depict the values of 〈*R*〉 obtained by continuation by parameter *τ* when it decreases and increases, respectively, starting from (A) *τ* = 180 ms and (B) *τ* = 150 ms. The horizontal dashed lines indicate the amount of synchronization in the stimulation-free STN. Parameters *g*_G→S_ = 1.7 nS/*μ*m^2^, pulse width *PW* = 0.5 ms, and the stimulation intensity (A) *K* = 10 and (B) *K* = 2 ⋅ 10^5^.

To further verify the robustness of the considered stimulation methods and to model the effect of slow variations of system parameters, we calculate the order parameter 〈*R*〉 of the STN neurons when the stimulation delay *τ* slowly changes. The initial conditions of the model for the next value of *τ* correspond to the last state of the system for the previous value of *τ*. The results of such a continuation by parameter *τ* are illustrated in Fig 13A for pulsatile LDF and in Fig 13B for pulsatile NDF. The variation of *τ*, which can correspond to the changes of the oscillation frequency of STN neurons, does not cause any problem with respect to the stimulation-induced desynchronization. Indeed, the pulsatile LDF stimulation with slowly varying delay [Fig 13A, red circles and blue squares] demonstrates the same desynchronizing effects as for the case where the stimulation is administered to initially synchronized population [Fig 13A, black solid curve]: The size and location of desynchronization regions are preserved. For the pulsatile NDF stimulation the situation can even be improved, where the variation of parameters significantly extends the desynchronization regions [Fig 13B, red circles and blue squares]. With such an approach of slowly varying stimulation parameters, the NDF stimulation can desynchronize the stimulated neurons for any value of the stimulation delay as has also been reported for other models [57, 61].

## Discussion

In this paper we showed that the delayed feedback methods preserve their desynchronizing impact on synchronized neurons also in the case when the smooth feedback stimulation signal is replaced by a high-frequency pulse train of charge-balanced pulses with amplitude modulated according to the corresponding feedback algorithm. This may pave the way to pre-clinical and clinical tests of the delayed feedback methods for counteracting abnormal neuronal synchronization in the framework of closed-loop DBS. The latter paradigm is in focus of research nowadays, as discussed in the introduction. For instance, in a particular, successfully tested aDBS approach [35], the stimulation voltage was linearly adapted to the patient’s beta band power each second. From a technical standpoint this is close to our approach, where the amplitude of the HF DBS pulse train is directly modulated by the delayed feedback signal [Fig 8]. Our results suggest that an appropriately processed and time-delayed adaptation of the stimulation amplitude to the beta power amplitude might further increase the stimulation outcome. We presented a detailed investigation and comparison of the properties of smooth and pulsatile LDF and NDF administered to a physiologically based model of STN-GPe neurons [71] contributing to the abnormally synchronized neuronal dynamics characteristic for Parkinson’s disease [8, 9, 18].

We showed that both smooth and pulsatile LDF and NDF techniques can robustly desynchronize the stimulated neurons and revealed the corresponding desynchronization regions in the parameter plane of the stimulation delay *τ* and stimulation intensity *K*. To address the possible problem mentioned by Dovzhenok *et al*. [73] that the feedback stimulation may be ineffective because of a possible variation of the extent of synchronization in neuronal populations, we calculated the desynchronization parameter regions for initially strongly and weakly (and intermittently) synchronized neurons. We showed that the desynchronization regions for initially weakly synchronized neurons do not differ much in size and location from those for initially strongly synchronized neurons, whereas the former can even be larger for an optimal range of the stimulation parameters. For smooth LDF, the desynchronization regions can be displaced by the delay parameter *τ* (especially for suboptimally large delay) with respect to each other for the above two cases of strong and weak initial synchronization [Fig 4]. This however is caused by a shift in the oscillation frequency and not by the extent of synchronization among neurons itself since the optimal value of the stimulation delay *τ* is related to the oscillation period as known from several publications [55, 56, 61]. In order to observe the effects reported by Dovzhenok *et al*. [73] for the optimal range of the stimulation parameters, one has to particularly search for the corresponding parameter values at the border of the desynchronization region, which significantly devalues the generality of the claims of Dovzhenok *et al*. [73]. For a practical application we hence need two types of closed-loop control processes: (i) On a fast time scale the measured signal is used for amplitude modulation of the HF pulse train according to the delayed feedback algorithm. (ii) On a slow time scale the delay of the feedback algorithm is adapted to the period of the abnormal oscillation in order to make sure the system is kept in a desynchronization region.

When parameters of the neuronal system significantly change to an extent considered by Dovzhenok *et al*. [73], the stimulation will in fact be administered to a completely different system with different dynamics, and the stimulation parameters have to be recalibrated. For the standard HF DBS, for example, selecting a clinically effective parameter set is an iterative process, and a systematic reprogramming of stimulation parameters is required in order to optimize clinical benefit [21–23, 78, 80, 91, 92]. Adaptive optimization of the stimulation parameters is a focus of research in the framework of the closed-loop DBS [78–80]. By the same token, for the delayed feedback stimulation characteristic stimulation parameters, especially the delay, should be adapted to measurable variables, such as the period of the abnormal oscillation. Using just one fixed set of stimulation parameters, irrespective of the system’s state, as suggested by Dovzhenok *et al*. [73], is neither an appropriate stimulation strategy nor does it take account of fundamental properties of delayed feedback stimulation mechanisms. The parameter adaptation for the delayed feedback stimulation can be done automatically as proposed in Ref. [64], which we successfully tested also for the considered STN-GPe model, see Fig 5. For smooth NDF as well as for pulsatile LDF and NDF, such a parameter recalibration seems to be unnecessary, where the stimulation-induced desynchronization obtained for initially strongly synchronized neurons is also preserved for initially weakly synchronized regime, see Figs 6 and 11. We therefore refute the conclusions of Dovzhenok *et al*. [73] for smooth NDF and do not confirm them for pulsatile LDF and NDF. Note, the authors of Ref. [73] do not distinguish between LDF and NDF which are clearly two different control techniques. Moreover, the desynchronizing impact of the pulsatile NDF is little sensitive to the stimulation delay when the latter slowly changes, which models the slow variations of the oscillation frequency [Fig 13B]. We also verified the efficacy of the considered approach for the case when neurons are perturbed by an independent noise modeling a noisy environment inevitable in real systems, see S2 Text. The investigated stimulation techniques are thus robust desynchronizing methods which can be promising candidates for closed-loop DBS.

For a successful utilization of any control method, the application conditions of the method have to be handled with care by precisely following all application requirements of a given method. Otherwise, any approach can easily be spoiled. The LDF and NDF techniques, for example, are essentially based on measuring of a mean field of a sufficiently representative, i.e., large enough oscillatory population such that the measured signal reliably reflects the properties of synchronized dynamics. The stimulation should also be administered to a large enough (sub)population with stimulation parameters appropriately related to the other parameters and properties of the stimulated system. All these fundamental requirements were not taken into account by the authors of Ref. [73]. By simulating a model containing 10 STN neurons during maximally 5 sec (less than 50 oscillation periods), measuring 5 or 3 neurons as a mean field (LFP) contaminated by the stimulation signal and stimulating only a few neurons with nonlinearly modified stimulation signal and suboptimally selected stimulation parameters, it was concluded that the delayed feedback “is unlikely to be clinically successful” [73] which is inappropriate and might, in fact, prevent other researchers from further fruitful theoretical investigations and pre-clinical as well as clinical tests of delayed feedback control methods.

However, due to the substantial stimulation-induced artifact [93], it could be difficult to perform the pulsatile LDF and NDF with the same stimulation and recording electrode. To remove the stimulation-induced artifacts, a special filtering has to be applied [78], or the stimulation and recording can be arranged by adjacent contacts in a specific bipolar configuration [34]. The pulsatile LDF and NDF can also be delivered by means of separate stimulation-registration setups [58, 63] or by an act-and-wait protocol [94].

The smooth LDF has been tested for many different models and stimulation setups demonstrating a pronounced desynchronizing effect [55, 56, 74]. The same has been demonstrated for smooth NDF [57, 61, 63]. The structure of the parameter space of LDF was also experimentally confirmed for arrays of coupled electrochemical oscillators [75]. The desynchronizing impact of smooth NDF was also confirmed experimentally for suppression of synchronized alpha-rhythm in visual cortex by visual stimulation in healthy subjects [76]. Several studies on closed-loop DBS revealed promising results [34, 35, 37–39, 77–79]. However, there are still relevant open questions [93, 95], and our approach, extending the LDF and NDF methods to physiologically relevant balanced pulsatile stimulation protocols, might help to resolve some of these issues. In particular, our approach requires an oscillatory biomarker sufficiently representing abnormal states or conditions. However, as yet, it is not clear whether such a biomarker is available [95]. For instance, so far it is not clear whether low or high frequency beta might be more appropriate as feedback signal [95]. Applying our method to different frequency (sub-)bands might help to probe and find possible oscillatory biomarkers. Actually, the beta oscillation alone might possibly not be an optimal feedback signal [93], e.g. because enhanced beta oscillations are not consistently observed in all Parkinson’s patients [52, 93]. For instance, characteristic changes of two distinct bands of high frequency oscillations (HFO), around 250 Hz and 350 Hz, were observed after levodopa administration, and the power ratio of these two bands was significantly correlated with the Unified Parkinson’s Disease Rating Scale hemibody akinesia/rigidity subscore, but not with beta power [96]. As suggested by this study (prokinetic) HFO (>200 Hz) and beta oscillations might potentially react in an independent manner to levodopa administration. The potentially differential response characteristics of HFO and beta oscillations to different types of aDBS still remain to be investigated. Another relevant aspect is patient phenotype [97]: In tremor dominant (as opposed to akinetic rigid) Parkinson’s patients resting state beta power might be reduced during epochs with tremor [53, 97]. In addition, low-frequency (theta and beta) and high-frequency oscillations interact under both physiological [98] and pathological [99] conditions. Physiological processes in motor circuits may be perturbed, since the HFO amplitude is coupled to the phase of beta oscillations [99, 100]. Hence, phase amplitude coupling (PAC) might be used as biomarker to trigger demand-controlled DBS [95]. Obviously, PAC-based closed-loop DBS does not easily translate to the feedback approach presented in our paper, since the latter requires a feedback signal representing the synchronized activity of a large neuronal population. Johnson *et al*. [87] performed another study demonstrating that STN beta LFP power alone will likely not be an appropriate biomarker. They compared traditional continuous DBS (tDBS) with closed-loop DBS (CL-DBS) in MPTP monkeys. CL-DBS reduced rigidity to an amount comparable to tDBS, but with approximately half the stimulation ON time. However, only tDBS improved bradykinesia during reaching tasks, probably because beta power was reduced related to the reaching process, in this way reducing the biomarker [87].

It would be interesting to compare the efficacy in suppressing pathological neuronal oscillations of the methods considered in this paper to other control methods, for instance, to those relying on event-based or phase-locked stimulation [66, 67, 101], where the stimuli are administered at a particular phase of the oscillation cycle. Vulnerable tremor phases were studied with non-invasive approaches [102, 103] as well as with thalamic DBS [104]. In the latter study [104] 10 patients with essential tremor were stimulated with low-frequency stimulation with thalamic electrodes, while tremor amplitude and phase were recorded. Stimulation with a stimulation frequency close to the postural tremor frequency caused a tremor entrainment. Tremor amplitude was modulated by the tremor phase at which the stimulus was delivered. Stimuli delivered in the favorable half of the tremor cycle caused a reduction of the median tremor amplitude by approximately 10%, whereas stimuli delivered in the opposite half of the tremor cycle caused an increase of the tremor amplitude by a similar amount. Stimuli administered at an optimal tremor phase caused a tremor reduction of 27%. Improvement in tremor severity by the standard HF DBS was on average 70%, supporting satisfactory DBS electrode placement. This approach might be further improved by taking into account the dependence on the tremor amplitude itself. According to the theory of phase resetting in noise-free [1] and noisy oscillatory populations [3, 30] it is known that the vulnerable phase of a synchronized neuronal population strongly depends on the extent of synchrony. If not properly adapted to the amount of synchrony, the single pulse may, in fact, massively boost synchrony [31].

In summary, as yet there is no an established biomarker representing the patients’ abnormal states or conditions [95]. In this context, we also have to take into account that increased beta power as well as PAC of beta phase to HFO amplitude may not be disease specific, but were also found in patients with isolated dystonia [86]. This may well limit the diagnostic value of these LFP features [86], but still they might be useful biomarkers for closed-loop DBS. However, applying our approach to different frequency (sub-)bands might eventually help to probe possible oscillatory candidates and help to reduce side effects by inducing long-lasting, sustained therapeutic effects by a specifically desynchronizing effect, as demonstrated in pre-clinical studies [48, 49] and a clinical proof of concept study [50] on CR-DBS.

## Conclusion

In this study we presented a novel method for demand-controlled desynchronization stimulation. To this end, we combined desynchronizing linear and non-linear delayed feedback stimulation techniques with cDBS by using the delayed feedback signal to modulate the amplitude of the cDBS pulse train. Such a stimulation signal satisfies mandatory safety requirements of charge deposit in neuronal tissue by electrical stimulation. In addition, it inherits the desynchronizing properties of the delayed feedback. We investigated the impact of smooth and pulsatile desynchronizing delayed feedback stimulation on a physiologically based model of a neuronal network of interacting STN-GPe neurons for linear and nonlinear delayed feedback techniques. We demonstrated that both feedback techniques can desynchronize both initially strongly and initially weakly synchronized STN neurons. When comparing smooth LDF and NDF stimulations, the latter appears to be less sensitive to parameter variations and stimulation conditions and has larger desynchronization parameter regions. The former, however, is more effective in inducing strong desynchronization, but less effective if desynchronization of a moderate extent is desirable. In the case of pulsatile stimulation, the difference between LDF and NDF is diminished. The pulsatile LDF is however hardly inducing synchronization for the considered range of parameters for initially weakly synchronized neurons, whereas the desynchronizing impact of pulsatile NDF is robust with respect to stimulation pulse shape and to slow parameter variations. Both stimulation techniques can be suggested for closed-loop desynchronizing DBS.

## Supporting information

S1 Appendix Parameters of the considered model of STN-GPe network. (PDF)

S1 Text Parameter selection for filtering by damped oscillator. (PDF)

S2 Text Impact of noise. (PDF)
