# Supplementary material for: Pulsatile desynchronizing delayed feedback for closed-loop deep brain stimulation
Source: PLoS One. 2017 Mar 8;12(3):e0173363. doi: 10.1371/journal.pone.0173363 (PMC5342235; doi:10.1371/journal.pone.0173363)
Supplement: S1 Appendix — (PDF) [file pone.0173363.s001.pdf]

S1 Appendix. Parameters of the considered model of STN-GPe network.

The values of parameters of the neuronal model (1) - (3) are listed in Table A. The model was originally introduced in paper [1], and the values of parameters mostly coincide with those from the papers [1–3] and are taken to demonstrate the synchronous bursting dynamics of the STN neurons, which is a hallmark of the pathological parkinsonian state [4, 5].

Table A. Parameter set of the considered model of STN-GPe network (1) - (3).

Parameter	STN	GPe	Units	Parameter	STN	GPe	Units
g_{L}	2.25	0.1	nS/μm^2	θ_m	-30.0	-37.0	mV
g_{K}	40	30	nS/μm^2	θ_h	-39.0	-58.0	mV
g_{Na}	50	120	nS/μm^2	θ_n	-32.0	-50.0	mV
g_{T}	0.5	0.5	nS/μm^2	θ_r	-67.0	-70.0	mV
g_{Ca}	0.5	0.15	nS/μm^2	θ_a	-63.0	-57.0	mV
g_{AHP}	9.0	30	nS/μm^2	θ_b	0.4	–	
v_{L}	-60.0	-55.0	mV	θ_s	-39.0	-35.0	mV
v_{K}	-80.0	-80.0	mV	θ_h^τ	-57.0	-40.0	mV
v_{Na}	55.0	55.0	mV	θ_n^τ	-80.0	-40.0	mV
v_{Ca}	140.0	120.0	mV	θ_r^τ	68.0	–	mV
τ_h^1	500.0	0.27	ms	θ_g^H	-39.0	-57.0	mV
τ_n^1	100.0	0.27	ms	θ_g	30.0	20.0	mV
τ_r^1	17.5	–	ms	σ_m	15.0	10.0	mV
τ_h^0	1.0	0.05	ms	σ_h	-3.1	-12.0	mV
τ_n^0	1.0	0.05	ms	σ_n	8.0	14.0	mV
τ_r^0	40.0	–	ms	σ_r	-2.0	-2.0	mV
ϕ_h	5	0.1		σ_a	7.8	2.0	mV
ϕ_n	5	0.3		σ_b	-0.1	–	
ϕ_r	2	1.0		σ_s	8.0	2.0	mV
k_1	15.0	30.0		σ_h^τ	-3.0	-12.0	mV
k_{Ca}	22.5	3.0		σ_n^τ	-26.0	-12.0	mV
σ_r^τ	-2.2	–	mV	σ_g^H	8.0	2.0	mV
α	5.0	2.0	ms^{-1}	β	1.0	0.045	ms^{-1}
$I_{\mathrm{app},j}$	$\mathcal{N}(10, 0.015^2)$	-4.24	pA/μm^2	τ_r	–	30	ms
C_m	1.0	1.0	pF/μm^2	ε_j	$5 \cdot 10^{-5}$	$\mathcal{N}(0.0055, [2 \cdot 10^{-5}]^2)$	ms^{-1}

References

1. Terman D, Rubin JE, Yew AC, Wilson CJ. Activity Patterns in a Model for the Subthalamopallidal Network of the Basal Ganglia. J Neurosci. 2002;22(7):2963–2976. doi:20026266.

2. Rubin JE, Terman D. High frequency stimulation of the subthalamic nucleus eliminates pathological thalamic rhythmicity in a computational model. J Comput Neurosci. 2004;16(3):211–235.

3. Park C, Worth RM, Rubchinsky LL. Neural dynamics in Parkinsonian brain: The boundary between synchronized and nonsynchronized dynamics. Phys Rev E. 2011;83(4):042901. doi:10.1103/PhysRevE.83.042901.

4. Hammond C, Bergman H, Brown P. Pathological synchronization in Parkinson's disease: networks, models and treatments. Trends Neurosci. 2007;30(7):357–364. doi:10.1016/j.tins.2007.05.004.

5. Benabid AL, Chabardes S, Mitrofanis J, Pollak P. Deep brain stimulation of the subthalamic nucleus for the treatment of Parkinson's diseaseEgge. Lancet Neurol. 2009;8(1):67–81. doi:10.1016/S1474-4422(08)70291-6.
