# Supplementary material for: Pulsatile desynchronizing delayed feedback for closed-loop deep brain stimulation
Source: PLoS One. 2017 Mar 8;12(3):e0173363. doi: 10.1371/journal.pone.0173363 (PMC5342235; doi:10.1371/journal.pone.0173363)
Supplement: S1 Text — (PDF) [file pone.0173363.s002.pdf]

S1 Text. Parameter selection for filtering by damped oscillator.

Time courses of the DC-balanced raw local field potential (LFP) measured as $LFP(t) = N^{-1} \sum_{j=1}^{N} s_j$, where $s_j(t)$ are the synaptic variables of STN neurons, are illustrated by black curves in Figs. 1A and 1B for the cases of strongly and weakly synchronized STN neurons, respectively. The corresponding filtered LFP (variable $\dot{x}(t)$ of the damped oscillator (5)) obtained by applying the linear damped oscillator with parameters $\alpha_d = k_f = 0.008$ and $\omega = 2\pi/T$, where T is the mean period of the raw LFP, are depicted by red curves in Figs. 1A and 1B, respectively. As one can see, for such parameters of the damped oscillator, the filtered LFP well follows the oscillations of the raw LFP in both cases of strongly and weakly synchronized STN neurons.

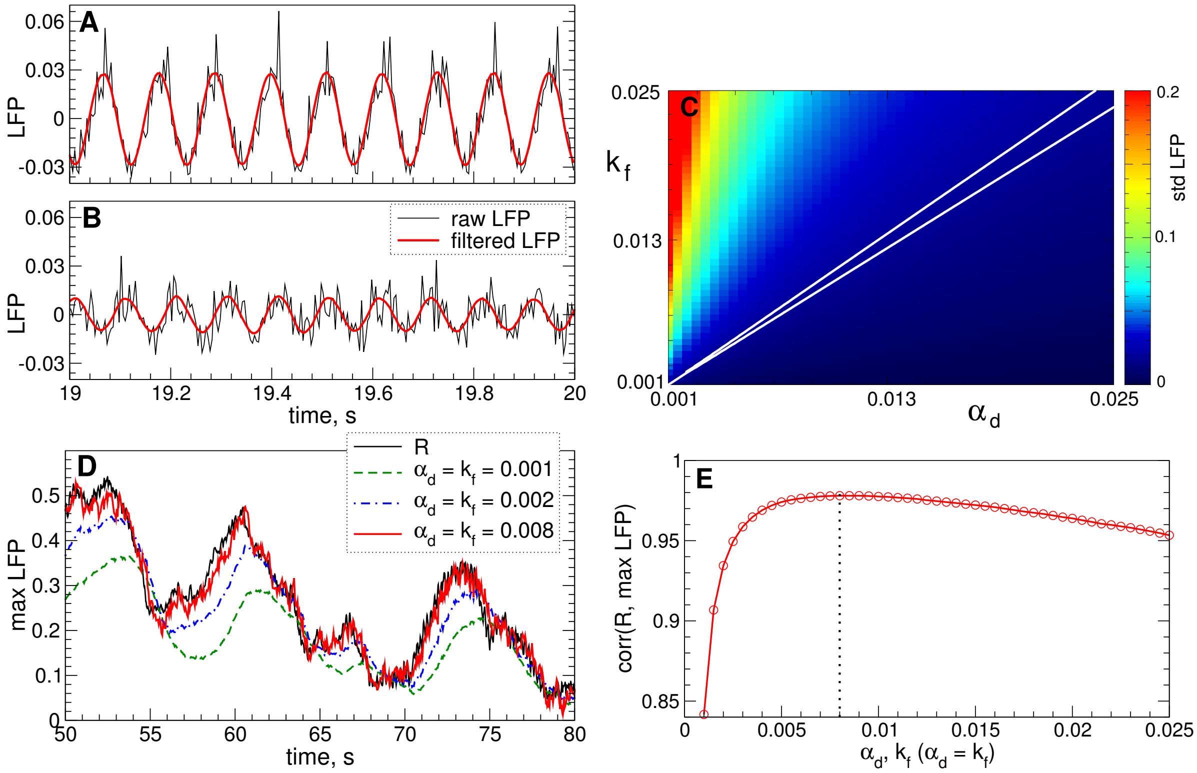

Figure 1. Parameter optimization of the linear damped oscillator for the on-line filtering of the STN LFP. (A), (B) Time courses of the DC-balanced raw LFP of (A) strongly and (B) weakly synchronized STN neurons are depicted by black curves. The corresponding LFPs filtered by the linear damped oscillator (5) with parameters $\alpha_d = k_f = 0.008$ and $\omega = 2\pi/T$, where T is the mean period of the raw LFP, are illustrated by red curves. (C) Standard deviation std LFP of the filtered LFP is depicted in color versus parameters α_d and k_f for the strongly synchronized regime. The white curves bound the parameter region, where $0.019 < \mathrm{std\,LFP} < 0.021$. (D) Time courses of the order parameter R (black curve) and local maxima max LFP of the rectified signal $|LFP|$ of the filtered LFP (scaled by the factor of 25) for parameters α_d and k_f indicated in the legend for the weakly synchronized regime. (E) Correlation between the order parameter R and max LFP from plot (D) versus parameters $\alpha_d = k_f$. Dotted vertical line indicates parameter value $\alpha_d = k_f = 0.008$. Parameters $T = 110$ ms and $g_{\mathrm{G}\to\mathrm{S}} = 1.7$ nS/μm^2 in plots (A) and (C), and $T = 100$ ms and $g_{\mathrm{G}\to\mathrm{S}} = 1.28$ nS/μm^2 in plots (B), (D) and (E).

To optimize the filtering outcome we scan the parameter plane (α_d, k_f) and calculate the standard

deviation std LFP of the filtered LFP for the case of the strongly synchronized regime [Fig 1A], which is depicted in color in Fig 1C. The white curves in Fig 1C bound the parameter region where $0.019 < \mathrm{std\,LFP} < 0.021$. For such parameters the standard deviation of the filtered LFP closely approaches the standard deviation of the raw LFP that was found to be approximately 0.02, see Fig 1A. As one can see, the parameter values $\alpha_d = k_f$ belong to the mentioned parameter region, and we thus consider such parameters as an optimal condition.

We verify the selected filtering parameters also for the considered case of weakly synchronized neurons [Fig 1B]. In such a regime, the order parameter R strongly fluctuates [Fig 1D, black curve], see also Fig 3C (red circles) of the paper. To compare the fluctuations of the order parameter with those of the amplitude of the filtered LFP, we extract the local maxima $\max \mathrm{LFP}(t)$ of the rectified signal $|LFP|$ of the filtered LFP and plot them (scaled by the factor of 25) in Fig 1D for parameters α_d and k_f indicated in the legend under the above optimal condition $\alpha_d = k_f$. Small values of the parameters result in a suboptimal filtering, where the amplitude of the filtered LFP does not precisely follow the fluctuations of the order parameter [Fig 1D, green and blue curves]. On the other hand, for larger filtering parameters, for instance, for $\alpha_d = k_f = 0.008$, the order parameter and the amplitude of the filtered LFP fluctuate nearly simultaneously, compare black and red curves in Fig 1D. Therefore, for such parameters the filtered LFP well represents the extent of synchronization in the neuronal population and can thus be used for the construction of the feedback stimulation signal. Further increase of the filtering parameters does not lead to a further significant improvement of the agreement between the order parameter and the amplitude of the filtered LFP.

To quantify the similarity between the time dynamics of the order parameter R and the amplitude of the filtered LFP, we calculate the correlation between $R(t)$ and $\max \mathrm{LFP}(t)$ [Fig 1D] and plot it in Fig 1E versus the filtering parameters α_d and k_f with $\alpha_d = k_f$. We found that the correlation grows as the filtering parameters increase and attains its maximum at $\alpha_d = k_f = 0.008$, where the order parameter and the amplitude of the filtered LFP strongly correlate [Fig 1E, dotted vertical line]. Any further increase of the filtering parameters can lead to decay of the correlation. We therefore consider the values $\alpha_d = k_f = 0.008$ as optimal filtering parameters and use them in our calculations.

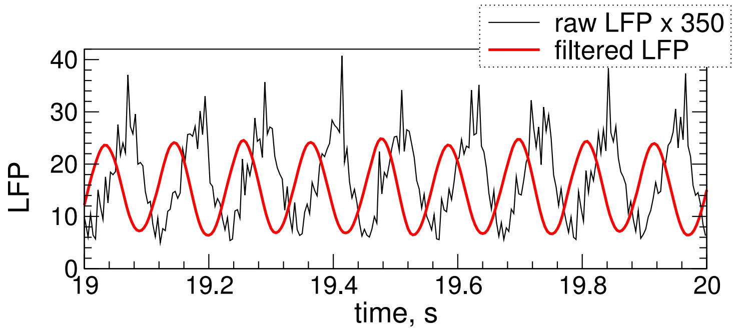

Figure 2. Filtering of the STN LFP by linear damped oscillator with suboptimal parameters. Time courses of the raw LFP (scaled by the factor 350) of strongly synchronized STN neurons and filtered LFP obtained with the use of the linear damped oscillator (5) with parameters $\alpha_d = \omega$, $k_f = 1$, and $\omega = 2\pi/T$ are depicted by black and red curves, respectively, as indicated in the legend. The filtered LFP is calculated as $LFP(t) = x(t - \tau_s)$, where $x(t)$ is the variable of the damped oscillator, and delay $\tau_s = 50\,\mathrm{ms}$. Parameter $g_{\mathrm{G}\to\mathrm{S}} = 1.7\ \mathrm{nS}/\mu\mathrm{m}^2$.

We also compare the selected optimal filtering parameters with those used by the authors of the paper [1], where $\omega = 2\pi/T$ as before, but $\alpha_d = \omega$ and $k_f = 1$. The delayed variable $x(t - \tau_s)$ of the

damped oscillator is used as the filtered LFP, where the delay $\tau_s = 50$ ms is taken to compensate for the phase shift caused by the filtering. We plot the raw and filtered LFP obtained for such filtering parameters in Fig 2 for the case of strongly synchronized STN neurons. Such a filtering approach leads to a filtered LFP that does not preserve the amplitude of the raw LFP and enhances it by a factor of approximately 350. Moreover, such a filtering does not remove the DC component of the raw signal and causes a large phase shift. Therefore, the filtering procedure utilized by the authors of Ref. [1] is not appropriate since it results in a signal whose properties strongly deviate from those of the measured raw LFP.

References

1. Dovzhenok A, Park C, Worth RM, Rubchinsky LL. Failure of Delayed Feedback Deep Brain Stimulation for Intermittent Pathological Synchronization in Parkinson's Disease. PLoS ONE. 2013;8(3):e58264. doi:10.1371/journal.pone.0058264.
