# Supplementary material for: Pulsatile desynchronizing delayed feedback for closed-loop deep brain stimulation
Source: PLoS One. 2017 Mar 8;12(3):e0173363. doi: 10.1371/journal.pone.0173363 (PMC5342235; doi:10.1371/journal.pone.0173363)
Supplement: S2 Text — (PDF) [file pone.0173363.s003.pdf]

S2 Text. Impact of noise.

To illustrate the impact of noise on the dynamics of STN and GPe neurons and stimulation-induced desynchronization by delayed feedback, we perturb the dynamics of the membrane potential v_i of neuron i by an additive independent noise $\mu\xi_i(t)$, where ξ_i is normally distributed with $\langle\xi_i\rangle = 0$ and $\langle\xi_i(t), \xi_j(t')\rangle = \delta(i-j)(t-t')$, and μ is the noise intensity. Without noise ($\mu = 0$), the neurons for the considered case of strong coupling ($g_{\mathrm{G}\to\mathrm{S}} = 1.7$ nS/μm^2) are synchronized with the order parameter $R \approx 0.69$. Noise of moderate intensity, e.g., $\mu = 0.06$, induces fluctuations of the order parameter, but the neurons still remain strongly synchronized with the time-averaged order parameter $\langle R\rangle \approx 0.66$ [Fig. 1A, B, red circles]. Stronger noise desynchronizes the neurons. We therefore consider the above noise intensity in order to demonstrate the desynchronizing impact of the feedback stimulation on a neuronal population perturbed by noise.

Time courses of the order parameter R of the noise-perturbed STN neurons stimulated by smooth linear delayed feedback (LDF) and nonlinear delayed feedback (NDF) are illustrated in Figs. 1A and 1B (black diamonds), respectively. As in the case without noise, the stimulation desynchronizes the neurons with noise, and the order parameter approaches small values in the course of the stimulation. The corresponding filtered LFPs and feedback stimulation signals of smooth LDF and NDF are illustrated in Figs. 1(C) and 1(D), respectively. The impact of the smooth LDF and NDF stimulations on the synchronized STN neurons with noise is illustrated in Figs. 1C and 1D, respectively, versus stimulation delay τ. The order parameter $\langle R\rangle$ for the noise-free case ($\mu = 0$) is also shown (red circles) for comparison. We found that noise of moderate intensity does not influence much the structure of the parameter space of the smooth delayed feedback stimulation. Both with moderated noise and without noise the regions of pronounced desynchronization (small values of $\langle R\rangle$) nearly coincide with each other for both smooth LDF and NDF stimulations [Fig. 1C, D]. This indicates the robustness of the reported results with respect to noise which is inevitable in real systems.

The same conclusion can also be drawn for the pulsatile feedback stimulation. We illustrate this in Fig. 2 for the cases of strong coupling [Fig. 2A, B] and weak coupling [Fig. 2C, D], where the STN neurons are initially strongly or weakly synchronized, respectively. The effect of the stimulation is qualitatively well preserved when the stimulated neurons are perturbed by noise of moderate intensity, compare the values of the order parameter in Fig. 2 for $\mu = 0$ and $\mu = 0.06$. In summary, the smooth and pulsatile LDF and NDF are robust desynchronizing stimulation techniques and can effectively induce desynchronization in strongly and weakly synchronized neuronal populations perturbed by noise.

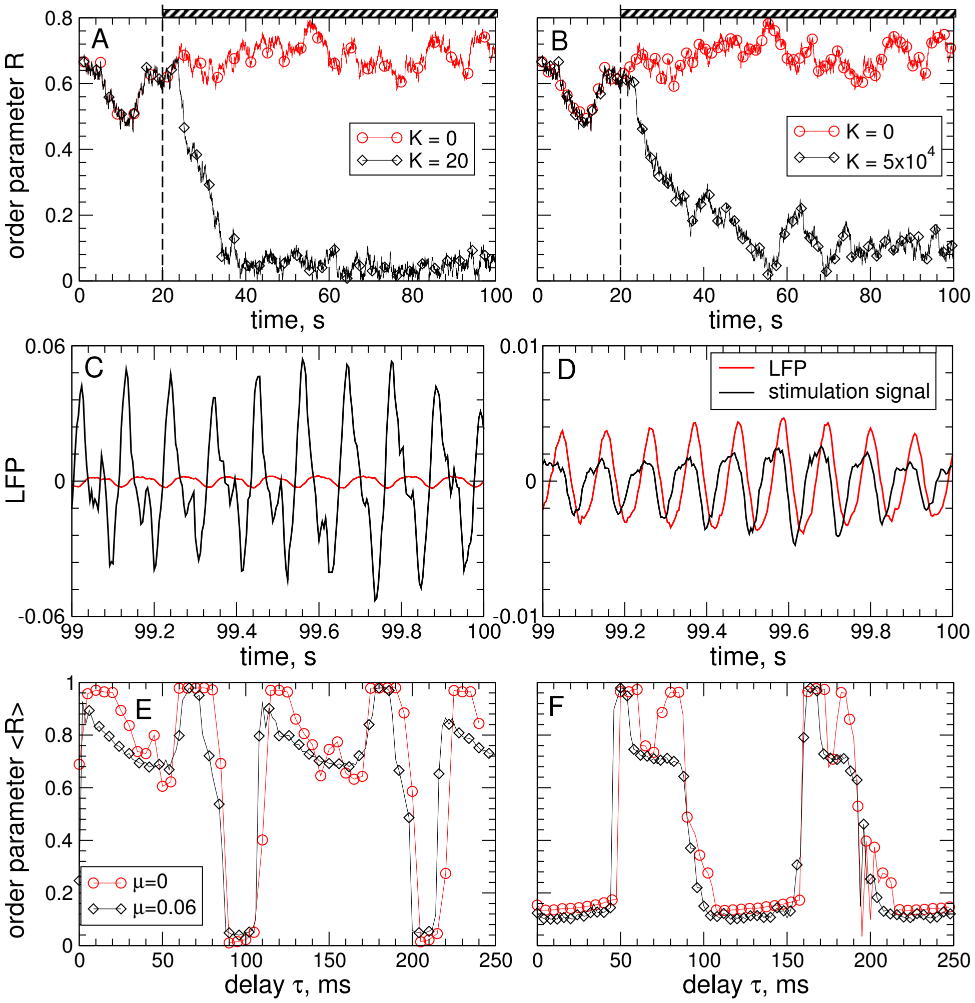

Figure 1. Control of synchronization of the noise-perturbed neurons by smooth LDF and NDF. (A), (B) Time courses of the order parameter R of STN neurons stimulated by (A) smooth LDF and (B) smooth NDF with intensities K indicated in the legends. Stimulation starts at time $t = 20$ s, and the stimulation epoch is indicated by black bar on the top of the plots. The order parameter of the STN neurons without stimulation ($K = 0$, red circles) is also shown. (C), (D) Time courses of the filtered LFP (red curves) and the corresponding feedback stimulation signals (black curves) of (C) smooth LDF and (D) smooth NDF from plots (A) and (B), respectively. (E), (F) The time-averaged order parameter $\langle R(t) \rangle$ of the STN neurons with noise of intensity μ indicated in the legends stimulated by (E) smooth LDF and (F) smooth NDF versus stimulation delay. Coupling parameter $g_{\mathrm{G}\to\mathrm{S}} = 1.7$ nS/μm^2, stimulation delay (A), (C) $\tau = 96$ ms and (B), (D) $\tau = 128$ ms, and the stimulation intensity (E) $K = 20$ and (F) $K = 5 \cdot 10^4$.

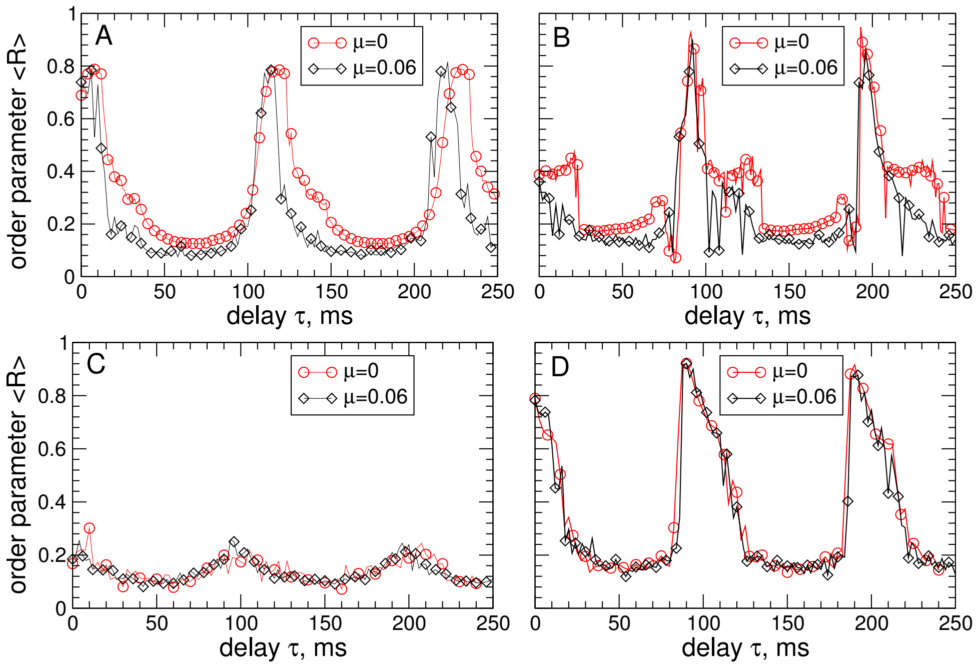

Figure 2. Control of synchronization of the noise-perturbed neurons by pulsatile LDF and NDF. The time-averaged order parameter $\langle R(t) \rangle$ of the stimulated STN neurons is plotted versus the stimulation delay τ for (A), (C) pulsatile LDF and (B), (D) pulsatile NDF stimulation. The corresponding stimulation signals consist of a pulse train of the charge-balanced biphasic pulses of type D with pulse width $PW = 0.5$ ms modulated by the LDF or NDF smooth signals, respectively. The neurons are perturbed by noise of the intensity μ indicated in the legends. The stimulation is administered to (A), (B) strongly coupled and (C), (D) weakly coupled STN neurons with $g_{\mathrm{G}\to\mathrm{S}} = 1.7$ nS/μm^2 and $g_{\mathrm{G}\to\mathrm{S}} = 1.28$ nS/μm^2, respectively. Stimulation intensity (A), (C) $K = 10$ and (B), (D) $K = 2 \cdot 10^5$.
